# Analysis of the Complete Genome Sequence of a Novel, Pseudorabies Virus Strain Isolated in Southeast Europe

**DOI:** 10.1155/2019/1806842

**Published:** 2019-04-04

**Authors:** Zsolt Csabai, Dóra Tombácz, Zoltán Deim, Michael Snyder, Zsolt Boldogkői

**Affiliations:** ^1^Department of Medical Biology, Faculty of Medicine, University of Szeged, Somogyi B. u. 4., Szeged H-6720, Hungary; ^2^Department of Genetics, School of Medicine, Stanford University, 300 Pasteur Dr., Stanford, CA 94305-5120, USA; ^3^Department of Biotechnology, Faculty of Science and Informatics, University of Szeged, Középfasor 52., Szeged H-6726, Hungary

## Abstract

**Background:**

Pseudorabies virus (PRV) is the causative agent of Aujeszky's disease giving rise to significant economic losses worldwide. Many countries have implemented national programs for the eradication of this virus. In this study, long-read sequencing was used to determine the nucleotide sequence of the genome of a novel PRV strain (PRV-MdBio) isolated in Serbia.

**Results:**

In this study, a novel PRV strain was isolated and characterized. PRV-MdBio was found to exhibit similar growth properties to those of another wild-type PRV, the strain Kaplan. Single-molecule real-time (SMRT) sequencing has revealed that the new strain differs significantly in base composition even from strain Kaplan, to which it otherwise exhibits the highest similarity. We compared the genetic composition of PRV-MdBio to strain Kaplan and the China reference strain Ea and obtained that radical base replacements were the most common point mutations preceding conservative and silent mutations. We also found that the adaptation of PRV to cell culture does not lead to any tendentious genetic alteration in the viral genome.

**Conclusion:**

PRV-MdBio is a wild-type virus, which differs in base composition from other PRV strains to a relatively large extent.

## 1. Background

Pseudorabies virus (PRV) also termed as Aujeszky's disease virus or suid alphaherpes virus 1 is the causative agent of Aujeszky's disease (AD) [1]. PRV is a herpesvirus belonging to species Suis alphaherpes virus 1, genus *Varicellovirus*, subfamily Alphaherpesvirinae, family Herpesviridae, order Herpesvirales. This virus has a broad host range including most mammalian animals and some avian species [2]. The natural reservoirs of the virus are the pig and the wild boar [3]. PRV causes considerable economic losses in the swine industry worldwide; therefore, programs for its eradication have been implemented in many countries [4]. Various live vaccines, such as Bartha-K61 [5], are utilized in these programs.

The adult swine is the only susceptible animal that can survive a PRV infection. The virus causes fatal encephalitis in all other susceptible species, including dogs, cats, laboratory mice, and rats, but these animals represent a dead-end for the PRV infection, because they are unable to transmit the disease through the conventional nasal route. Humans are resistant to PRV. Among others, these features make PRV an ideal model organism for studying the molecular biology and pathomechanism of herpesviruses [6, 7]. Furthermore, this virus is widely used as a live tracer for mapping polysynaptic neural circuits [8–10] for gene delivery to cardiac muscle cells [11] and neurons [12], as well as for oncolytic virotherapy [13]. PRV has also been utilized as a model for the investigation of transcriptional interference networks, which is supposed to be a novel universal regulatory layer of gene expression [14].

The life cycle of PRV is mainly controlled at the level of transcription. Herpesvirus genes are expressed in a coordinated, cascade-like fashion [15] and are traditionally divided into three major temporal classes in terms of their peak rates of mRNA synthesis and their behavior in the presence of protein or DNA synthesis inhibitors: immediate-early (IE), early (E), and late (L). The IE genes encode transcriptional activators; the E gene products represent the synthetic machinery of DNA replication; the L proteins form the structural elements of the virus.

Until now, 23 full-length PRV DNA sequences have been described. The viral genome is composed of two unique regions, the unique long (UL) and the unique short (US) segments. The US is bracketed by inverted repeat (IR) sequences, but the two ends of the UL region are only partially homologous to each other, which indicates that these parts of the genome were homologous in the ancestor of this virus, such as in the herpes simplex virus (HSV). The PRV genome exists in four isomeric forms [16]. The PRV genome encodes at least 67 protein-coding genes, 20 noncoding transcripts, and a large number of polycistronic RNAs, as well as transcript isoforms, including splice and transcript end variants [17]. The dynamic transcriptome of PRV was also characterized by real-time RT-PCR [18] and by single-molecule real-time (SMRT) sequencing [19] developed by Pacific Biosciences (PacBio). The PacBio RSII platform is the first commercially available third-generation sequencer, which is able to determine thousands of very long, single-DNA molecules in parallel and in real-time without amplification [20, 21]. The major importance of the long reads in the genome sequencing is that it is able to span of repetitive elements and to resolve them, which makes the reconstruction and assembly of the genomes easier [22]. Compared to the short-read sequencing, an important feature of the long-read sequencing (LRS) technique is that it results in unbiased coverage regardless of GC content. Thus, using the PacBio LRS method is particularly useful for the sequencing of small genomes with high GC contents and with many repetitive sequences [23]. The PRV genome is composed of very high overall GC content (∼74%) and a large number of repetitive elements; therefore, it is difficult to sequence it with the new generation short-read techniques or with the Sanger method. PacBio sequencing is advantageous compared to other techniques in that it does not produce systematic errors and any that may arise can be easily corrected due to its high consensus accuracy [24].

## 2. Results

### 2.1. Clinical Signs

The first clinical signs in infected piglets 5–14 days of age were fever, listlessness, and anorexia which were then quickly followed by tremors, seizures, or other signs of CNS involvement. Some piglets with hind leg paralysis sit on their haunches. Mortality at this age was 92 percent, and the affected piglets usually died within 24 to 36 hours. Sudden death could also be witnessed. Similar signs occurred in weaning pigs, but the mortality rate was lower than 18 percent. Vomiting and respiratory signs could be seen in older groups, finishing pigs, and sows. Postmortem lesions were serous to fibrinonecrotic rhinitis and exudative keratoconjunctivitis. In the central nervous system, leptomeningeal hyperemia could be seen. Affected pigs had necrotic tonsillitis and small (1–3 mm) necrotic foci occurred in the liver and spleen.

### 2.2. Isolation of Pseudorabies Virus from the Organs of Infected Pigs

**Figure 1:**
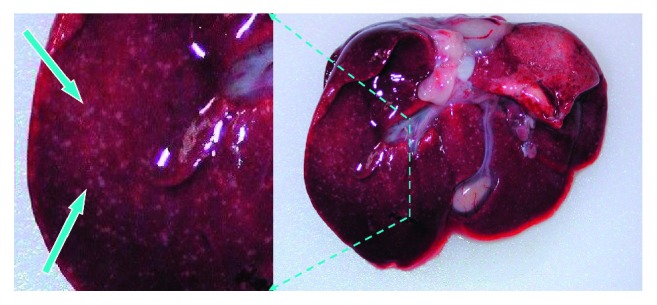
Lesions in the liver of an infected piglet. The pathological lesions on the liver of the dead piglet show typical characteristics to that of PRV infection. Blue-white arrows point to these multiple small white spots which are scattered randomly on the organ surface. The left side of the picture is a magnification of the picture on the right side; the area is surrounded by light-blue dashed lines.

**Figure 2:**
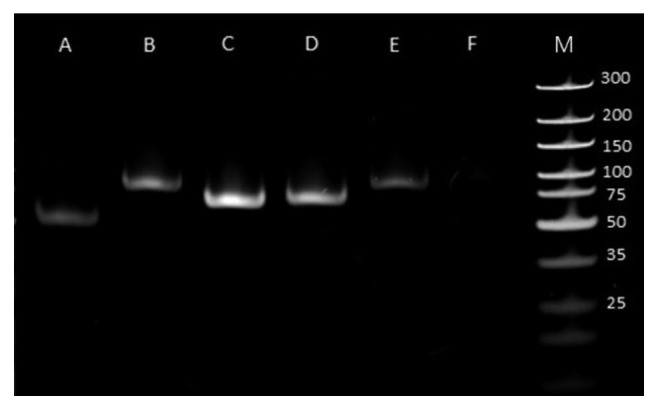
Detection of PRV from brain samples by PCR analysis. The amplification products of PCR analysis have been proven to be specific to the PRV DNA. A: *ul21*; B: *ul23*; C: *ul29*; D: *ul44*; E: *ul23* PRV-Kaplan (positive control); F: *ul23* PK-15 Cell line (negative control); M: molecular weight marker (Thermo Scientific™ GeneRuler™ Ultra Low Range DNA); the primer pairs used in this study is enlisted in Additional file 1. The amplicon lengths are as follows: *ul21*: 51 bp; *ul23*: 81 bp; *ul29*: 66 bp; *ul44*: 70 bp.

The infected organs have been dissected from a dead pig in a PRV outbreak occurred several years before the genetic analysis. The clinical symptoms of AD are not specific; therefore, we examined the infected organs (liver and spleen) of the pigs prior to the detailed molecular analysis. We observed pathological lesions that are typical of PRV infection (Figure 1). We also carried out neutralization tests using PRV-specific antibodies to confirm that this virus was responsible for the observed symptoms. The test showed a complete inhibition of the appearance of cytopathic effect on cultured porcine-kidney-15 (PK-15) cells. Additionally, regular PCR analysis was performed to verify the results of the neutralization test. All of the four primer pairs (Additional file 1) produced PRV-specific amplification products (Figure 2).

### 2.3. Cytopathic Effect

**Figure 3:**
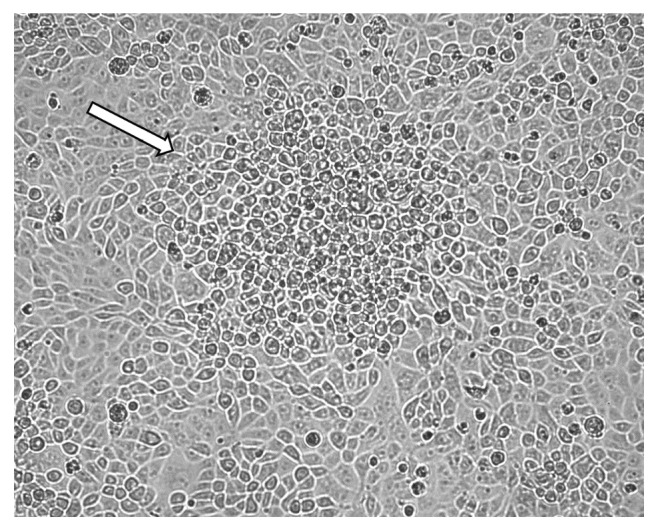
Cytopathic effect of PRV-MdBio in cultured PK-15 cells. Strain MdBio of PRV induces cytopathic effect on the immortalized PK-15 cells within 18 h after infection. The rounded cells (shown by the white arrow) indicate a plaque formed by infected cells.

We analyzed the cytopathic effect exerted by PRV-MdBio on immortalized PK-15 cells. The infection by the new strain produced typical rounded cells in the viral plaques by 18 h after infection (Figure 3), which was similar to those of normally observed in wild-type (*wt*) PRVs.

### 2.4. Growth Properties of Strain MdBio of PRV

**Figure 4:**
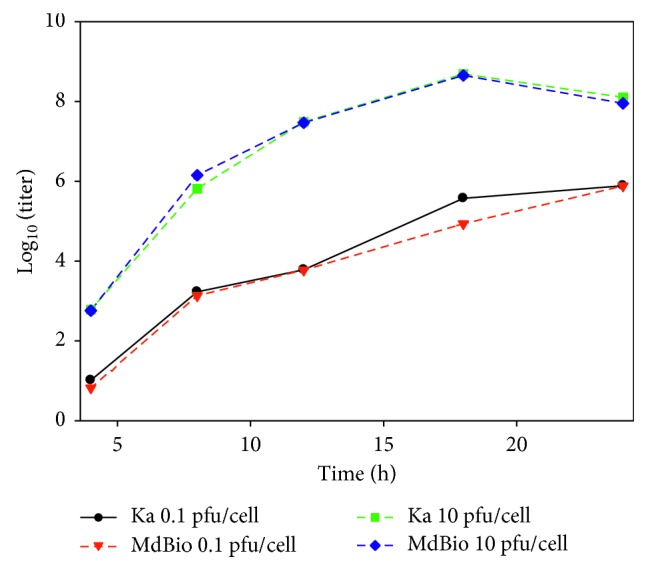
Growth curves of PRV-MdBio and PRV-Ka. The growth properties of two PRV strains were compared using both low (MOI = 0.1 pfu/cell) and high (MOI = 10 pfu/cell) titer of infection. The growth curves proved to be very similar, which indicates that PRV-MdBio is a wt virus.

We compared the growth characteristics of the novel viral strain with those of PRV-Ka using both low (MOI = 0.1 pfu/cell) and high dose (MOI = 10 pfu/cells) of viral infections. As a result, we obtained that the two *wt* viruses exhibited very similar growth properties in both experiments (Figure 4, Additional file 2). In the high MOI infection experiment, the maximal virion production occurred at 18 h after infection for both viruses, while it was maximal at 24 h after infection in the low MOI experiments.

### 2.5. Statistics of the Raw Data and Determination of the Nucleotide Composition of PRV-MdBio Using the PacBio Long-Read Sequencing Platform

**Figure 5:**
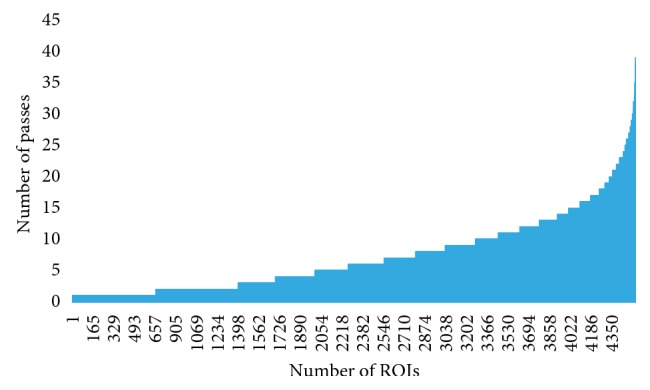
The count of aligned ROI reads (*x* axis) versus the number of subreads (*y* axis). About 8% of the reads contain only a single subread (these are generally the longer (>5 kb) reads), whereas about 10% of the ROIs builds up from at least 15 subreads. The highest number of passes is 39.

**Figure 6:**
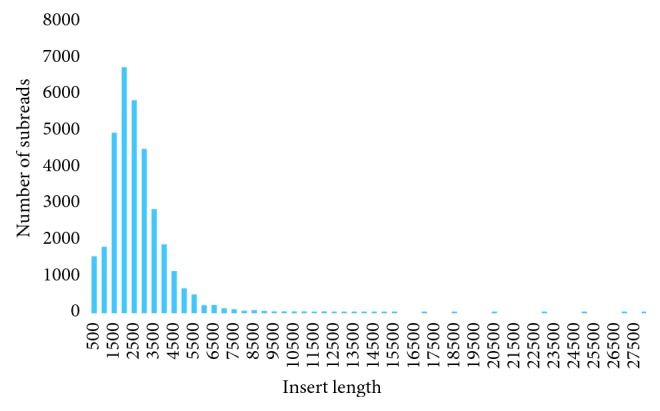
Length distribution of inserts shown for 500 base pair long bins. The bar chart illustrates the lengths of the subreads (insert between two SMRTbell adapter sequences) versus the number of subreads. The average length is 2324 bp.

In this study, the PacBio LRS technique was carried out for the determination of the base composition of the PRV. The RSII sequencing generated 32,768 raw subreads, which resulted in 4,450 high-quality read of inserts (ROIs; 7.36 full passes on average; Figures 5 and 6).

The quality (the QC value) of the subreads varies between 0.750 and 0.901, with an average of 0.870, which means that the error rate is between 9.9% and 25% in our data (Table 1). The mean of the error rate is 13.4%, which is between of the previously published values (11 to 15%) [20, 25, 26].

**Figure 7:**
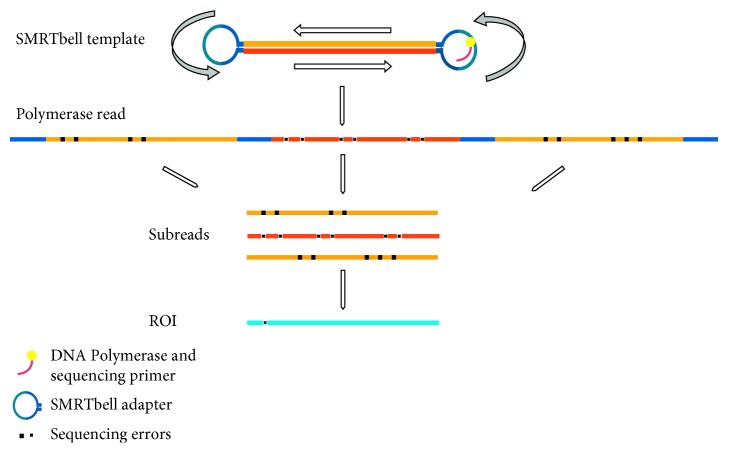
Schematic representation of a SMRTbell template sequencing. The figure shows the sequencing process and the read terminology. It can be seen that the inserts will be read many times: the complete sequence, including the adapters, is called as polymerase read. After the adapter sequences are removed, the sequence is split into subreads. The circular consensus read (CCS), also known as read of insert (ROI), is the high-quality consensus sequence from subreads that are from the same insert.

Regardless of the relatively high error rate for the subreads of the RSII sequencer, one of the outputs from the PacBio platform is the ROI (previously termed as circular consensus sequence) read [27]. ROI is an error-corrected consensus read derived from the alignment of subreads belonging to the same single circular template (SMRTbell), which can be sequenced multiple times in a single run (Figure 7) [20].

The quality of the ROIs was very high, according to the length of the subreads and the passes (it was approximately 99%).

The analysis of the obtained sequences revealed that strain MdBio is composed of 142,922 nucleotides, with a 73.56% GC content. We observed a relatively low-level polymorphism within the isolated MdBio strain (Table 2).

#### 2.5.1. Differences between the PRV-MdBio and PRV-Ea Strains

**Figure 8:**
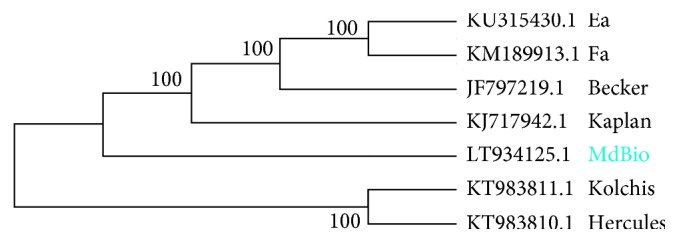
Evolutionary analysis by maximum likelihood method. The evolutionary tree was generated using whole-genome sequences. This analysis involved 7 nucleotide sequences. There were a total of 148,466 positions in the final dataset.

We demonstrated that the nucleotide composition of strain Ea of PRV (KU315430) isolated in China exhibits the greatest difference compared to strain MdBio (Figure 8.). Phylogenetic analysis—based on the full-genome sequences—of six previously reported PRV strains (Kaplan: KJ717942.1; two Greek strains: Hercules: KT983810.1 and Kolchis: KT983811.1; two Chinese strains: Ea KU315430.1; and Fa KM189913.1; as well as the Bartha strain: JF797217.1.) indicates that one of the closest relative of MdBio is the well-characterized [17–19] strain Kaplan. This is a widely used laboratory model strain, which was sequenced by the same LRS method [28].

**Figure 9:**
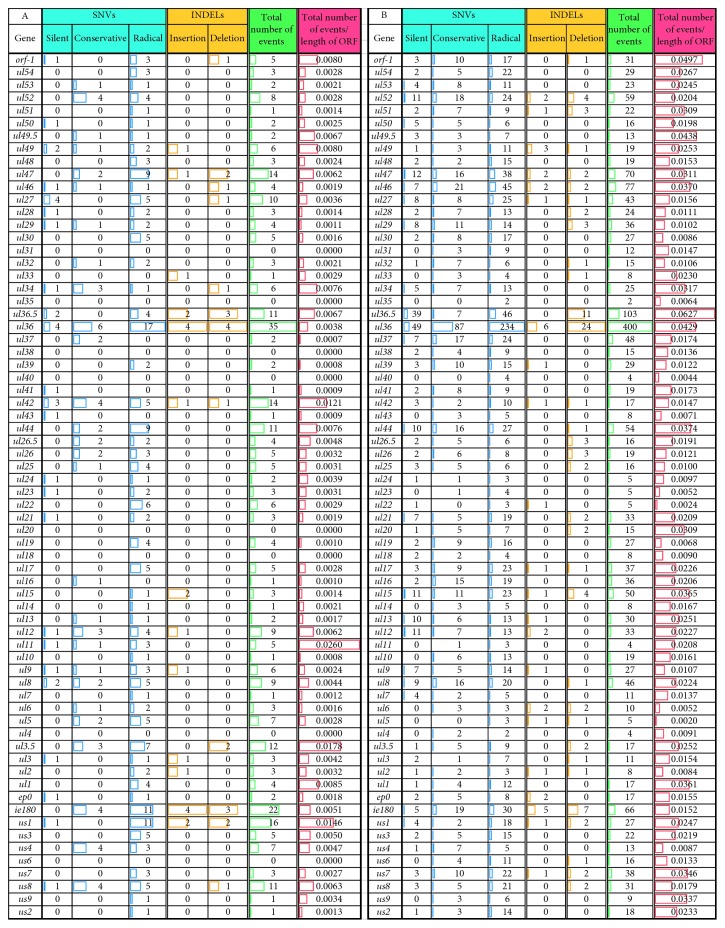
Comparison of the number of SNVs and INDELs within the open reading frames of PRV genes between the MdBio and two other strains. (a) The number of mutations between the MdBio and Ea strains was analyzed. This picture shows the exact numbers of the occurring synonymous, conservative, and radical SNVs, along with the insertions and deletions, the total numbers of the genetic mutation events, as well as their relative frequency compared to a given sequence length. Horizontal bar graphs represent their number. Blue color was used for labeling the number of SNVs within the ORFs, orange for the INDELs, while the red represents the number of global events. Additionally, the relative frequency of mutations was labeled by green. (b) The number of SNVs and INDELs within the open reading frames between the MdBio and Kaplan strains was analyzed. This picture—containing horizontal bars—shows the numbers of the detected single-nucleotide variation, insertions and deletions, the total numbers of the mutation events, and the number of mutations normalized with the lengths of coding sequences. The horizontal bar chart shows their numbers. Blue color was used for labeling the number of synonymous, conservative, and radical base replacements within the ORFs, orange for the insertions and deletions, while the red shows the number of global events. Furthermore, the relative frequency of mutations is shown in green.

The differences in base composition revealed by *in silico* analysis are shown in Table 3. We identified 2027 single-nucleotide variations (SNVs) within the open reading frames (ORFs) of the PRV genes. The SNV variations represent 268 synonymous, 519 conservative, and 1,109 radical changes. The radical SNVs are the most frequent point mutations in all PRV genes, except the *ul32* and *us4* genes, where there are more conservative changes (Figure 9(a)). We have also detected 140 INDEL variants within 38 ORFs (Table 3, Figure 9(a)). The ORFs of the following genes contain both insertions and deletions: *ul52*, *ul51*, *ul49*, *ul47*, *ul46*, *ul27*, *ul36*, *ul42*, *ul17*, *ul15*, *ul6*, *ul5*, *ul2*, *ie180*, *us7*, and *us1* (Figure 9(a)). The highest number of mutation events occurred in the *ul36* gene, whereas most mutations happened in the *ul36.5* gene (embedded into *ul36*) if we normalized the number of mutations with the length of coding sequences (CDS) (Figure 9(a)). Interestingly, there are only two mutation events within the ORF of *ul35* gene, which is oppositely oriented to the *ul36*/*ul36.5* gene cluster. The least number of mutations per nucleotide had occurred in the *ul5* gene (Figure 9(a)). Furthermore, the CDSs of 29 PRV genes contain only point mutations (SNVs), whereas the rest of the CDSs have both INDELs and SNVs (Figure 9(a)). Beside this, we also analyzed the intergenic regions, where we identified 1817 SNVs, as well as 396 INDELs (Table 3). Our analysis identified altogether 4381 mutation events between the genomes of the two examined PRV strains.

#### 2.5.2. Comparison of PRV-MdBio with Strain Kaplan Propagated Long-Term in Cell Culture

We also compared strain MdBio isolated from infected pigs and strain Kaplan (KJ717942.1) [28], which was propagated in cultured PK-15 cells for many years. We were interested in whether adaptation to cell culture results in any tendentious alterations within the viral genome. As for the point mutations in the ORFs, we obtained 307 SNVs representing 34 synonymous, 65 conservative, and 212 radical changes (Table 4). The number of INDELs in ORFs is 50, affecting 18 genes. The *ul33* gene is exceptional, because it contains only an insertion and there are no SNVs within its ORF. Note that an insertion in a strain may actually be a deletion in another strain, and this is true for the deletions.

There are both insertions and deletions in the protein-coding part of the *ul47*, *u42*, *ul36, us1* and and *ie180* genes (Figure 9(b)). The ORFs of 17 PRV genes have point mutations and INDELs, while the rest of the genes (41 genes) have only SNVs. Our data show that most mutation events occurred in the *ul36* gene (35 independent changes), but the frequency of mutations per base pair is the highest in the CDS of the *ul11*. No nucleotide alterations are present in the CDSs of the following eight genes: *ul31*, *ul35*, *ul38*, *ul40*, *ul20*, *ul18*, *ul4*, and *us6* (Figure 9(b)). In the intergenic regions, we detected 638 SNVs and 245 INDELs. We have identified seven genes that were affected by radical nucleotide substitution in their known protein domains, which were as follows: *us1, us3, ul13, ul15, ul22, ul27,* and *ul43.*

We found a much higher polymorphism in strain Ka than in strain MdBio, the reason for which may be that the pigs are usually infected by a small number of viral particles, which provides a bottle neck effect that reduces the genetic variance. This is not the case with strain Ka, which was propagated for a long time in immortalized cells, using a relative high titer of infection for each passage, and furthermore the cultured cells represent much more relaxed selective environment than the living organism having an immune system.

#### 2.5.3. Differences between the MdBio, Kaplan, and Ea Strains

**Figure 10:**
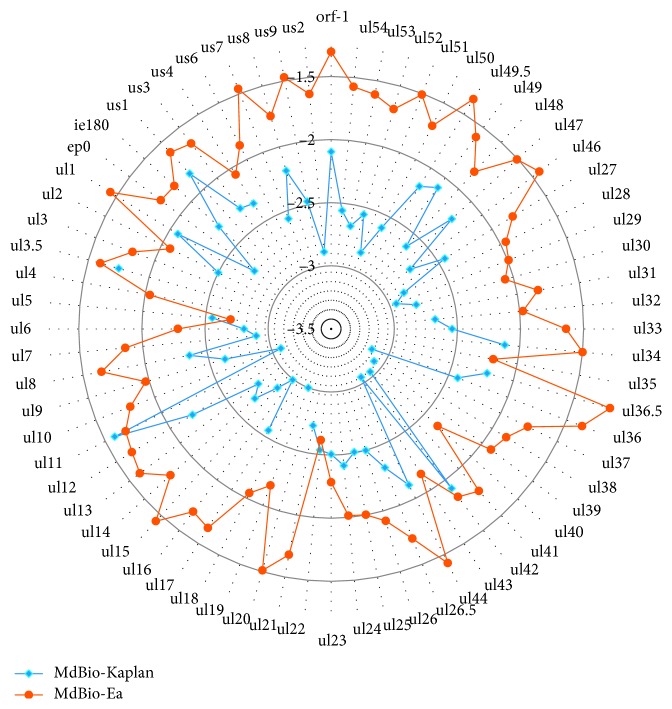
The relative mutation ratio within the genes compared to their length. The polar plot indicates the number of mutations in two PRV strains compared to PRV-MdBio per unit nucleotide sequence using log_10_ scale.

The general differences between the above three PRV strains were also determined. Comparing the total number of mutation events that occurred within a given nucleotide length, we found that the difference is much higher between the MdBio and Ea strains than between the MdBio and Kaplan viruses, except the above three genes: *ul22*, *ul11*, and *ul5* (Figure 10). The frequency of mutations is more than 20-fold between the MdBio and the Ea strains than between the MdBio and Ka strains for the following genes: *ul15* (27-fold), *ul37* (24-fold), *ul51* (22-fold), and *ul16* (20-fold) (Figure 10, Additional file 3). Furthermore, the *ul36* gene has the largest number of mutation events in each mutation types (Figure 9, Additional file 4) between the MdBio-Ka and MdBio-Ea strains, but when we normalized the number of mutations with the CDS lengths, the following data were obtained: the ORF with the highest number of SNVs was the *ul11*, as well as the *ul36.5* between the MdBio-Ka and MdBio-Ea, respectively, while the most INDEL mutation happened in the *us1* and *ul36.5* genes (Additional file 4, panel B). The number of SNV alterations is higher between the MdBio and Ea strains than between the MdBio and Ka in all CDSs, except for the following: *ul42*, *ul23*, *ul22*, *ul11*, *ul6*, *ul5*, *ul3.5*, *us1*, and *us4*.

Additionally, comparing the number of INDELs, it can be seen that the ORFs of *ul34* and *us1* have more difference between the MdBio and Ka than between the MdBio and Ea strains. Analysis of our results also shows that the same number of mutations occurred between the MdBio-Ka and MdBio-Ea pairs in six viral genes (two INDELs within the *ul42* and *ul3.5* and one insertion or deletion in the *orf-1*, *ul33*, *ul9*, and *ul3*), while there are no INDEL variations within the ORFs of 28 genes (Additional file 4).

**Figure 11:**
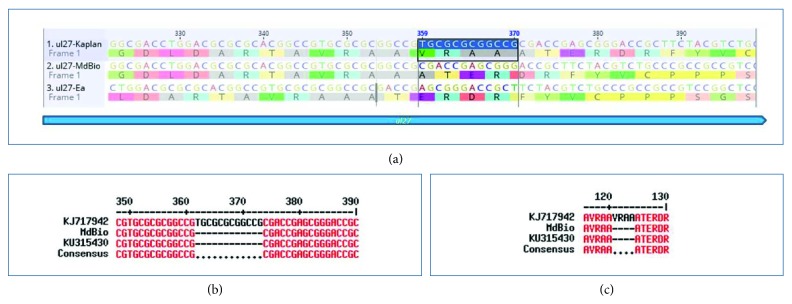
Length isoforms of ul27 gene. The ul27 gene of PRV-Ka is represented in two isomeric forms, a long and a short variant, which is in contrast to PRV-MdBio and Ea, which contain only the short length isomer. (a) Geneious alignment of the affected region of the *ul27* gene of the three PRV strains. (b) Sequence alignment of a short part of the *ul27* gene in the examined virus strains by Multaline. (c) Amino acid blast of the *ul27* gene among the MdBio, Kaplan, and Ea strains.

The *ul27* gene of strain Kaplan contains a variation at genomic position 19310–19321/19322–19333 (numbers represent the locations of the first and second repeat units). The short- and long-length variants differ from each other in a 12-nucleotide (TGC GCG CGG CCG) stretch encoding the V-R-A-A tetrapeptide, which is missing from the shorter isoform of this gene (Figure 11). The strain MdBio and Ea have no such variation; they only contain the short isoform.

Furthermore, we compared GC content of the three examined PRV strains, but no significant difference (0.04%) was found between them, ranging from 73.56% (GC content of MdBio) to 73.60% (GC content of Ea). The GC distribution of the strain Kaplan is 73.59%.

**Figure 12:**
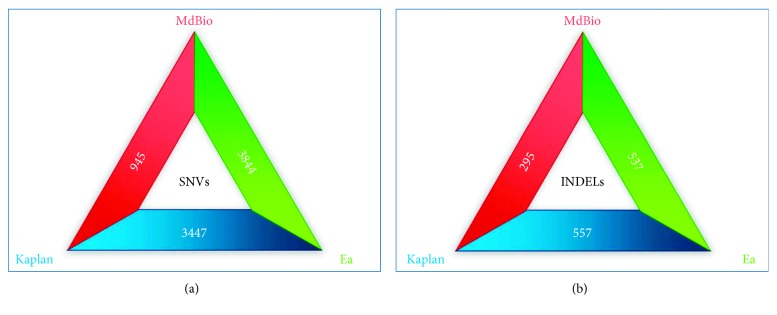
The general differences between the MdBio, Kaplan, and Ea strains of PRV. The total number of single-nucleotide variants (SNVs) (a) and insertions and deletions (INDELs) (b) between the three virus strains is represented by using triangle diagrams.

The general differences between the above three PRV strains is illustrated by using triangle representation (Figure 12). The genome-wide sequence alignments of the viral genomes were generated by Geneious software platform ([29]; Additional file 5 and 6).

## 3. Discussion and Conclusions

In this work, we isolated and characterized a novel PRV strain termed MdBio using the PacBio RSII LRS platform. This technique allowed us to easily assemble the complete genome (∼143 kbps in length with nearly 74% GC content), including the repetitive regions. PacBio RSII sequencing is more costly and results in lower coverage than most other methods; however, the importance of high-quality long reads for the genome assembly surpasses its disadvantages. This technology is superior over the short-read technologies for sequencing small genomes, such as viral DNAs.

The MdBio virus strain behaves similar to another *wt* virus (strain Ka of PRV) in that it is able to cause AD in pigs, and it exhibits growth curve on cultured cells comparable to the *wt* Kaplan strain. However, the base composition of strain MdBio differs considerably from other PRV strains including PRV-Kaplan to which it exhibits high similarity and to PRV-Ea to which it exhibits the least similarities. Intriguingly, a largest number of mutations within the ORFs were found to be radical, and less frequent mutations were silent in both strains that were compared to PRV-MdBio, which is not the case in the higher-order organisms. The reason for this peculiarity of PRV evolution continues to remain unexplained.

In recent years, the genomes of several virulent PRV strains have been sequenced (e. g., in Greece, China, and Italy). According to our analysis, the strain Ka is one of the closest relatives of our novel PRV isolate. Although the number of mutations between strain MdBio and Ka are relatively large, these mutations do not appear to affect the virulence.

Aujeszky's disease widely occurs, and outbreaks have been reported in many pig farms in Serbia during the last decades [30–32].

PRV circulates worldwide in wild boar populations, which seems to be an important reservoir of this virus. National eradication programs have resulted in that AD virtually disappearing from domestic pigs in several European countries, as well as in Canada, New Zealand, and the United States [33]; however, it does not necessarily mean complete eradication of the virus. Despite the successful elimination of the virus from domestic pigs, PRV infections have been reported year-on-year in various European countries [2,3,34–44] in wild boar, wolf, red fox, Iberian lynx, and in hunting dogs. Aujeszky's disease has also been reported in wild boar in Serbia [45]. It can be seen that, despite the eradication and vaccination efforts, potential viral transfer may occur through contact with wildlife.

## 4. Methods

### 4.1. Virus Isolation

Aujeszky disease occurred in ninety-eight sow herds in North Serbia, Kelebia town. The herds were closed and were free from leptospirosis, brucellosis, and Porcine Reproductive and Respiratory Syndrome (PRRS). Aujeszky's disease appeared in this farm seven years ago.

### 4.2. PCR Analysis

To confirm the identity of the isolated virus, the infected cell culture was analyzed by PCR technique. We used five primer pairs for the PCR reaction, which is shown in Additional file 1.

### 4.3. Cells and Virus Propagation

Immortalized porcine kidney epithelial (PK-15) cells were maintained at 37°C in the presence of 5% CO_2_. Cells were grown in DMEM (Gibco/Thermo Fisher Scientific), supplemented with 5% fetal bovine serum (Gibco/Thermo Fisher Scientific) and 80 *μ*g of gentamycin per ml (Gibco/Thermo Fisher Scientific). PK-15 cells were used for the propagation of pseudorabies virus. For the preparation of virus stock solution, PK-15 cells were infected with a low multiplicity of infection (MOI; 0.1 plaque-forming units (pfu)/cell). Viral infection was allowed to proceed until complete cytopathic effect was observed. Viruses were released from the infected cells by three successive cycles of freezing and thawing.

### 4.4. Virus Neutralization

For further identification of the virus, the supernatant of the infected PK-15 cell culture was used in the neutralization test with PRV neutralizing antibody to PRV. All samples were examined with PRV commercial ELISA kits (IDEXX Laboratories). A standard infectious dose of PRV was incubated for 1 hour at 37°C with 25% of the serum. Afterwards, this virus-serum mixture was inoculated on PK-15 cells. The inoculum was removed after 1 hour, and cell cultures were washed 2 times, new cell medium was added, and cultures were maintained at 37°C in the presence of 5% CO_2_. After 36 hours of incubation, cells were fixed and stained for PRV antigens using an immunoperoxidase technique. The number of infected cells was counted and compared to the number of infected cells in a PK-15 culture inoculated with a PRV stock treated with the serum of the pig before inoculation.

### 4.5. Isolation of Virions and Viral DNAs

For the isolation of virions, PK-15 cells were infected with PRV-MdBio using MOI = 0.1 pfu/cell and then incubated until complete cytopathic effect was observed. Virions were separated from the PK-15 cells by ultracentrifugation (Sorwall WX Ultra 90, Thermo Scientific) on a 30% sucrose cushion as has been described earlier [46]. The viral DNA was extracted from the samples using the traditional phenol/chloroform method.

### 4.6. Pacific Biosciences Single-Molecule Real-Time Sequencing

A SMRTbell template was prepared from the isolated viral DNA as previously described [47, 48], using standard protocols for 5 kb library preparation (PacBio “Procedure and Checklist—5 kb Template Preparation and Sequencing”). For the preparation of genomic library, 2 *μ*g DNA was sheared by using g-TUBEs (Covaris) according to the manufacturer's recommendations. Briefly, the DNA sample was diluted to 150 *µ*l, it was centrifuged at 11.000 × g for 30 sec, then the tube was inverted, and the sample was centrifuged again, with the same settings. The fragmented sample was concentrated using the PacBio AMPure PB magnetic beads (0.45x volume). The sheared and concentrated DNA was subjected to the “Repair DNA Damage” step, following the abovementioned protocol and using the appropriate components from the PacBio® Template Prep Kit: DNA damage repair buffer, NAD+, ATP high, dNTP, and DNA damage repair mix were added to the sample, and then the mixture was incubated at 37°C for 20 minutes. The next stage is the “Repair Ends” at 37°C for 20 using the end repair mix from the kit. The sample was purified using the PB beads, and it was followed by the “Blunt Ligation reaction”: the end-repaired sample was mixed with blunt adapter, and they were mixed. Template prep buffer and ATP low were added to the sample, then after a brief mixing, the ligase (all from the template prep kit) was added. The reaction was carried out at 25°C for 15 minutes, and then the enzyme was inactivated at 65°C for 10 min. To remove the failed ligation products, ExoIII and ExoVII exonucleases were added to the sample after the ligation. Sample was purified with the PB beads (three purification steps followed each other).

Annealing and binding conditions of sequencing primers and polymerase to the purified SMRTbell Template were calculated using the binding calculator (Pacific Biosciences). The DNA/Polymerase Binding Kit P6 v2 (Pacific Biosciences) was used for the annealing and SMRTbell Template binding. The sequencing primer was diluted in elution buffer (150 nM), and it was mixed with the sample and the primer buffer. The annealing was performed at 20°C for 2 h. The DNA polymerase was diluted (50 nM) using the PacBio Binding Buffer v2, and then it was bound to the annealed template. This sample complex was added to washed MagBeads (PacBio), and then they were incubated in a HulaMixer (Life Technologies) at 4°C for 30 min. After this binding step, the sample was purified with binding buffer then with wash buffer. The final elution was in 19 *μ*l binding buffer. The MagBead-bound sample complexes were loaded for sequencing.

Sequencing was carried out on the PacBio RSII long-read sequencing instrument (Pacific Biosciences) taking one 240-min movie for a single SMRT Cell with P6 DNA polymerase and C4 chemistry (P6-C4), yielding a total of 4,450 viral reads and a high coverage (∼70×) across the genome.

### 4.7. Data Analysis

Sequencing reads were aligned to the PRV-Ka reference genome (KJ717942.1) using the BLASR long-read aligner (PacBio). Integrative Genomics Viewer (IGV) [49] was used for data visualization and mapping quality assurance. Artemis (Wellcome Trust Sanger Institute) [50] was also used for the visualization and analysis of the sequencing data. Reads were de novo assembled using Geneious 10.1.3 software [29]. The MultAlin, online multiple sequence alignment tool was also used for visualization [51].

The evolutionary history was inferred by using the maximum likelihood method and general time reversible model [52]. The bootstrap consensus tree inferred from 500 replicates [53] is taken to represent the evolutionary history of the taxa analyzed [53]. Branches corresponding to partitions reproduced in less than 50% bootstrap replicates are collapsed. The percentage of replicate trees in which the associated taxa clustered together in the bootstrap test (500 replicates) are shown next to the branches [53]. Initial tree(s) for the heuristic search were obtained automatically by applying Neighbor-Join and BioNJ algorithms to a matrix of pairwise distances estimated using the maximum composite likelihood (MCL) approach and then selecting the topology with superior log likelihood value. A discrete Gamma distribution was used to model evolutionary rate differences among sites (5 categories (+G, parameter = 0.0500)). The rate variation model allowed for some sites to be evolutionarily invariable ([+I], 0.00% sites). Evolutionary analyses were conducted in MEGA X [54].

## Figures and Tables

**Table 1:** This table contains the quality data of the obtained subreads.

Statistics	Values
Mean	0.866
SD	0.022
Median	0.871
Mode	0.875
Max.	0.901
Min.	0.750

**Table 2:** Genetic polymorphism within strain MdBio of PRV.

Position start	Position end	Length	Change	Coverage	Polymorphism type	Variant frequency (%)	*P* value
2 690	2 712	23	GAGAGGAGATGGGGAGAGGAGAT	41 ≥ 43	Deletion	41.9 ≥ 43.9	7.40E-09
2 713	2 724	12	(GGGAGAGGAGAT)3 ≥ (GGGAGAGGAGAT)2	39 ≥ 41	Deletion (tandem repeat)	48.8 ≥ 51.3	3.30E-10
24 258	24 258	1	C ≥ T	80	SNV (transition)	26.30	5.70E-24
101 041	101 041	1	C ≥ T	123	SNV (transition)	27.60	2.90E-08

Our detailed analysis revealed that there are two insertion/deletion polymorphisms, as well as two polymorphic SNVs in the novel PRV strain.

**Table 3:** Genetic differences between strain MdBio and strain Ea of PRV.

Type of variants	CDS	Total number of cases	Detailed numbers of different mutations	
SNV	−	1817	Conservative	519
	+	2027	Radical	1109
			Synonymous	268

INDEL	−	396	Insertions	211
			Deletions	185
	+	140	Insertions	45
			Deletions	95

This Table contains the list of genetic differences between strains MdBio and Ea. CDS: coding sequence; SNV: single-nucleotide variant; INDEL: insertion and deletion.

**Table 4:** Genetic differences between strain MdBio and strain Kaplan of PRV.

Type of variants	CDS	Total number of events	Detailed numbers of different mutations	
SNV	−	638	Conservative	65
	+	307	Radical	212
			Synonymous	34

INDEL	−	245	Insertions	104
			Deletions	141
	+	50	Insertions	26
			Deletions	24

This table contains the list of genetic differences between strains MdBio and Kaplan. CDS: coding sequence; SNV: single-nucleotide variant; INDEL: insertion and deletion.

## Data Availability

The sequence data were submitted to the European Nucleotide Archive (ENA) with the accession number LT934125.1 (available at https://www.ebi.ac.uk/ena/data/view/LT934125).

## Abbreviations

AD: Aujeszky's disease
CDS: Coding sequence
E: Early
HSV: Herpes simplex virus
IE: Immediately-early
INDEL: Insertion and deletion
L: Late
PacBio: Pacific Biosciences
PK-15: Porcine-kidney-15 epithelial cell line
ORF: Open reading frame
PRV: Pseudorabies virus
RT-PCR: Reverse-transcription polymerase chain reaction
SMRT: Single-molecule real-time
SNV: Single-nucleotide variant
UL: Unique long
US: Unique short.

## Supplementary Materials

Supplementary 1 Additional file 1: the primers used for the PCR-identification of PRV.

Supplementary 2 Additional file 2: comparison of the growth properties of PRV-MdBio and PRV-Ka. In order to compare the growth properties of strains MdBio and Kaplan of PRV, we carried out a growth experiment using three independent biological replicates for the infections at each time point. Viral growth was analyzed using low (MOI = 0.1) and high (MOI = 10) dose of infection.

Supplementary 3 Additional file 3: bar chart representation of the number of mutations within the coding sequences. This graph shows the number of mutations in strains Ka and Ea of PRV compared to PRV-MdBio.

Supplementary 4 Additional file 4: horizontal chart representation of the number of SNVs and INDELs within the MdBio and Kaplan, and the MdBio and Ea strains. This horizontal bar chart indicates the total number of the SNVs and INDELs, which are different between the PRV-MdBio and two other viral strains (strains Ka and Ea). Panel A: blue color was used for labeling the number of SNVs within the ORFs and magenta for the INDELs. Panel B: blue color represents the normalized number of SNVs within the ORFs, while magenta was used for labeling the INDELs.

Supplementary 5 Additional file 5: pairwise sequence alignment of the MdBio strain and its furthest relative. This figure shows the MdBio and Ea sequence alignment. The genome sequences are represented by grey. The single-nucleotide variants are labeled by vertical, thin black lines within the genome. The thick black regions within the genome sequences are the insertions, whereas the deletions are represented by horizontal black lines. The protein-coding sequences of the genes are indicated by blue arrows. The histogram above the genomes shows the differences between the two sequences by yellow, while the identical parts are labeled by green.

Supplementary 6 Additional file 6: sequence comparison of the strain MdBio and Kaplan. This figure shows the sequence alignment of the strain MdBio and Kaplan. The genome sequences are represented by grey. The single-nucleotide variants are labeled by vertical, thin black lines within the genome. The thick black regions within the genome sequences are the insertions, whereas the deletions are represented by horizontal black lines. The protein-coding sequences of the genes are indicated by blue arrows. The histogram above the genomes shows the differences between the two sequences by yellow, while the identical parts are labeled by green.
