# Supplementary material for: Analysis of the Complete Genome Sequence of a Novel, Pseudorabies Virus Strain Isolated in Southeast Europe
Source: Can J Infect Dis Med Microbiol. 2019 Apr 4;2019:1806842. doi: 10.1155/2019/1806842 (PMC6476139; doi:10.1155/2019/1806842)
Supplement: Supplementary 1 — Additional file 1: the primers used for the PCR-identification of PRV. [file 1806842.f1.doc]

| **Name** | **Sequence (5'-3')** |
| --- | --- |
|  |  |
| **ul23 fw** | ATGACGGTCGTCTTTGACCGCCAC |
| **ul23 rev** | CGCTGATGTCCCCGACGATGAA |
| **ul29 fw** | CTGATCCTGCGCTACTGCG |
| **ul29 rev** | ACTGCATCGTGATCCCCG |
| **ul44 fw** | TCGTGAGCAGCATGATCGT |
| **ul44 rev** | GTCGCCATGATGACCAGC |
| **ie180 fw** | CATCGTGCTGGACACCATCGAG |
| **ie180 rev** | ACGTAGACGTGGTAGTCCCCCA |
