# Supplementary material for: Analysis of the Complete Genome Sequence of a Novel, Pseudorabies Virus Strain Isolated in Southeast Europe
Source: Can J Infect Dis Med Microbiol. 2019 Apr 4;2019:1806842. doi: 10.1155/2019/1806842 (PMC6476139; doi:10.1155/2019/1806842)
Supplement: Supplementary 2 — Additional file 2: comparison of the growth properties of PRV-MdBio and PRV-Ka. In order to compare the growth properties of strains MdBio and Kaplan of PRV, we carried out a growth experiment using three independent biological replicates for the infections at each time point. Viral growth was analyzed using low (MOI = 0.1) and high (MOI = 10) dose of infection. [file 1806842.f2.doc]

| **Time** | **Ka 0.1 pfu/cell** | | **MdBio 0.1 pfu/cell** | | **Ka 10 pfu/cell** | | **MdBio 10 pfu/cell** | |
| --- | --- | --- | --- | --- | --- | --- | --- | --- |
|  | **Mean** | **SE** | **Mean** | **SE** | **Mean** | **SE** | **Mean** | **SE** |
| **4h** | 1.03E+01 | 8.39E-01 | 6.67E+00 | 1.64E+00 | 6.07E+02 | 3.89E+01 | 5.73E+02 | 4.86E+01 |
| **8h** | 1.70E+03 | 3.48E+02 | 1.35E+03 | 1.96E+02 | 6.50E+05 | 1.30E+05 | 1.41E+06 | 5.20E+05 |
| **12h** | 6.07E+03 | 5.40E+02 | 5.97E+03 | 7.46E+02 | 3.07E+07 | 4.76E+06 | 2.93E+07 | 3.02E+06 |
| **18h** | 3.73E+05 | 1.19E+05 | 8.60E+04 | 4.84E+03 | 4.90E+08 | 5.78E+07 | 4.50E+08 | 7.37E+07 |
| **24h** | 7.77E+05 | 1.04E+05 | 7.67E+05 | 7.13E+04 | 1.26E+08 | 2.13E+07 | 8.93E+07 | 3.02E+06 |
