# Supplementary figures and images for: Analysis of the Complete Genome Sequence of a Novel, Pseudorabies Virus Strain Isolated in Southeast Europe
Source: Can J Infect Dis Med Microbiol. 2019 Apr 4;2019:1806842. doi: 10.1155/2019/1806842 (PMC6476139; doi:10.1155/2019/1806842)

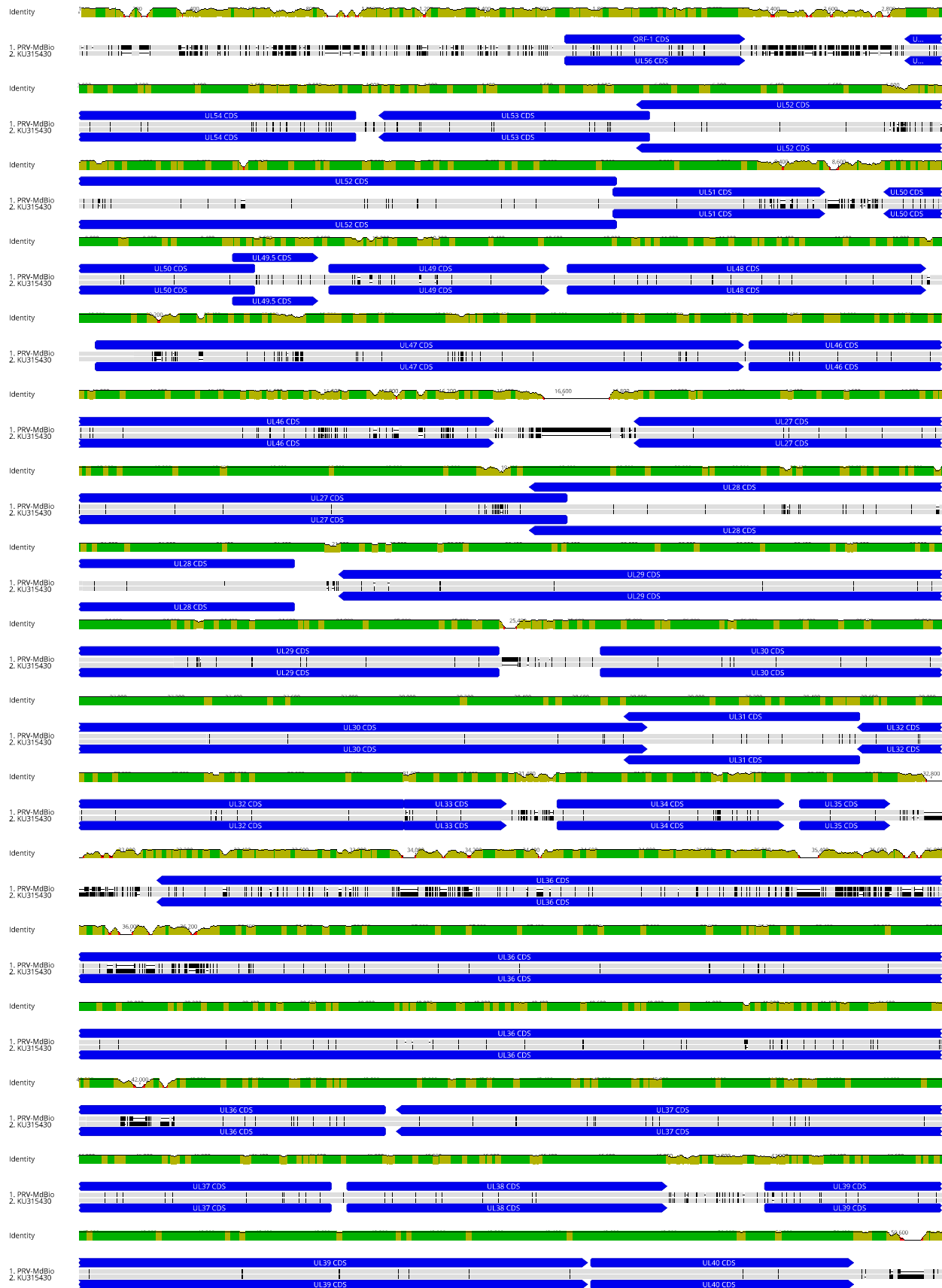

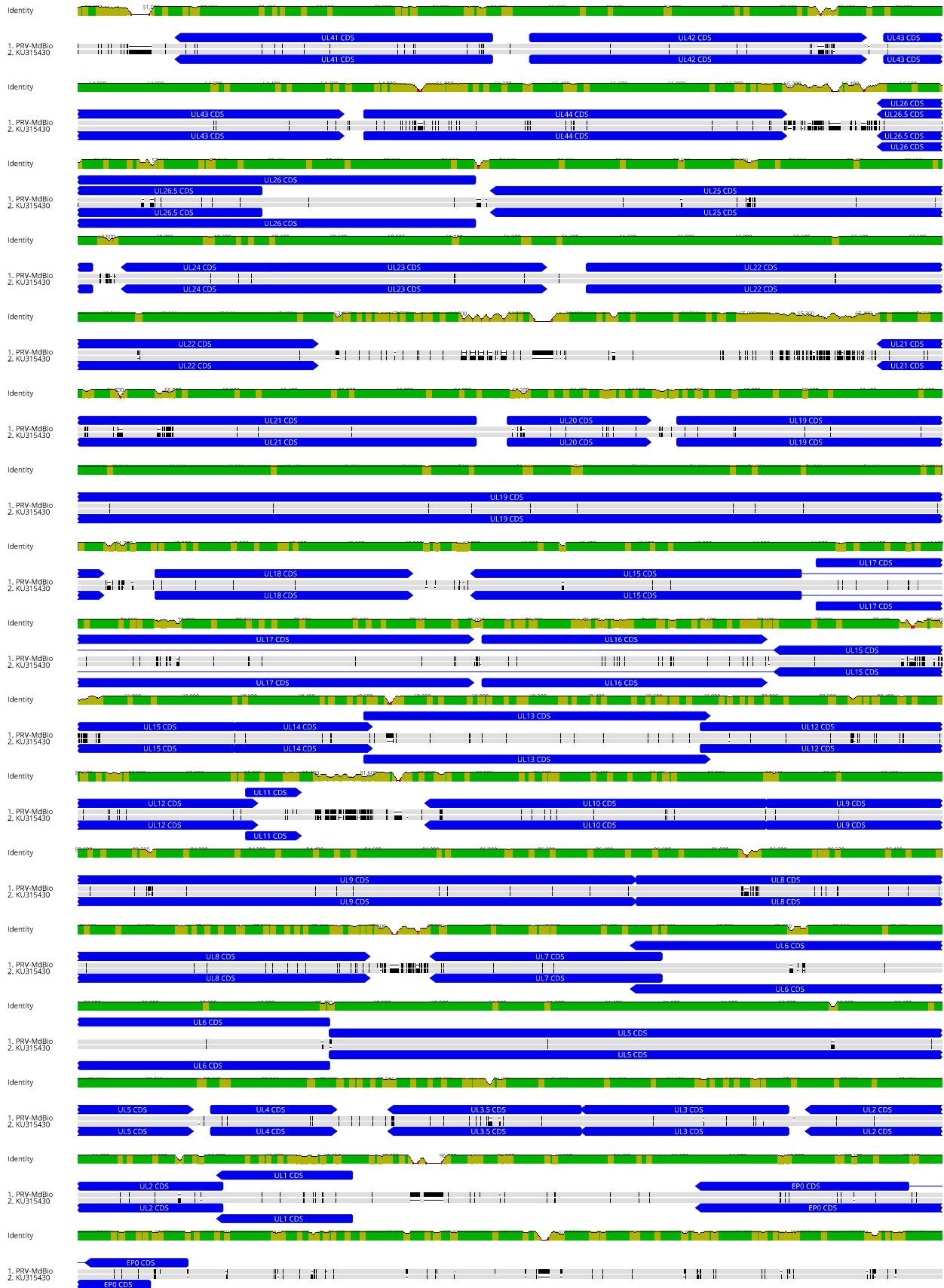

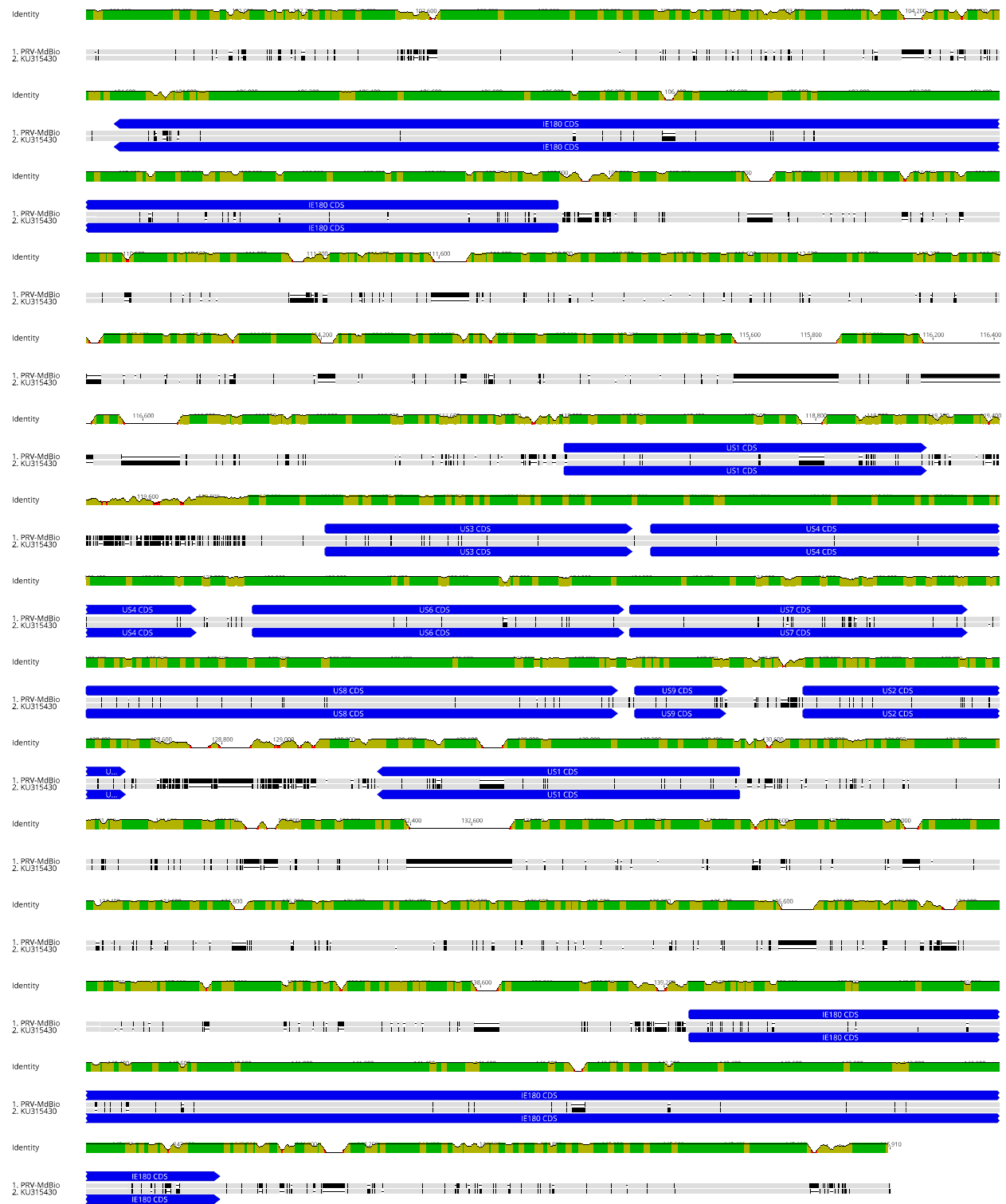

Supplement: Supplementary 3 — Additional file 3: bar chart representation of the number of mutations within the coding sequences. This graph shows the number of mutations in strains Ka and Ea of PRV compared to PRV-MdBio. [file 1806842.f3.pdf]

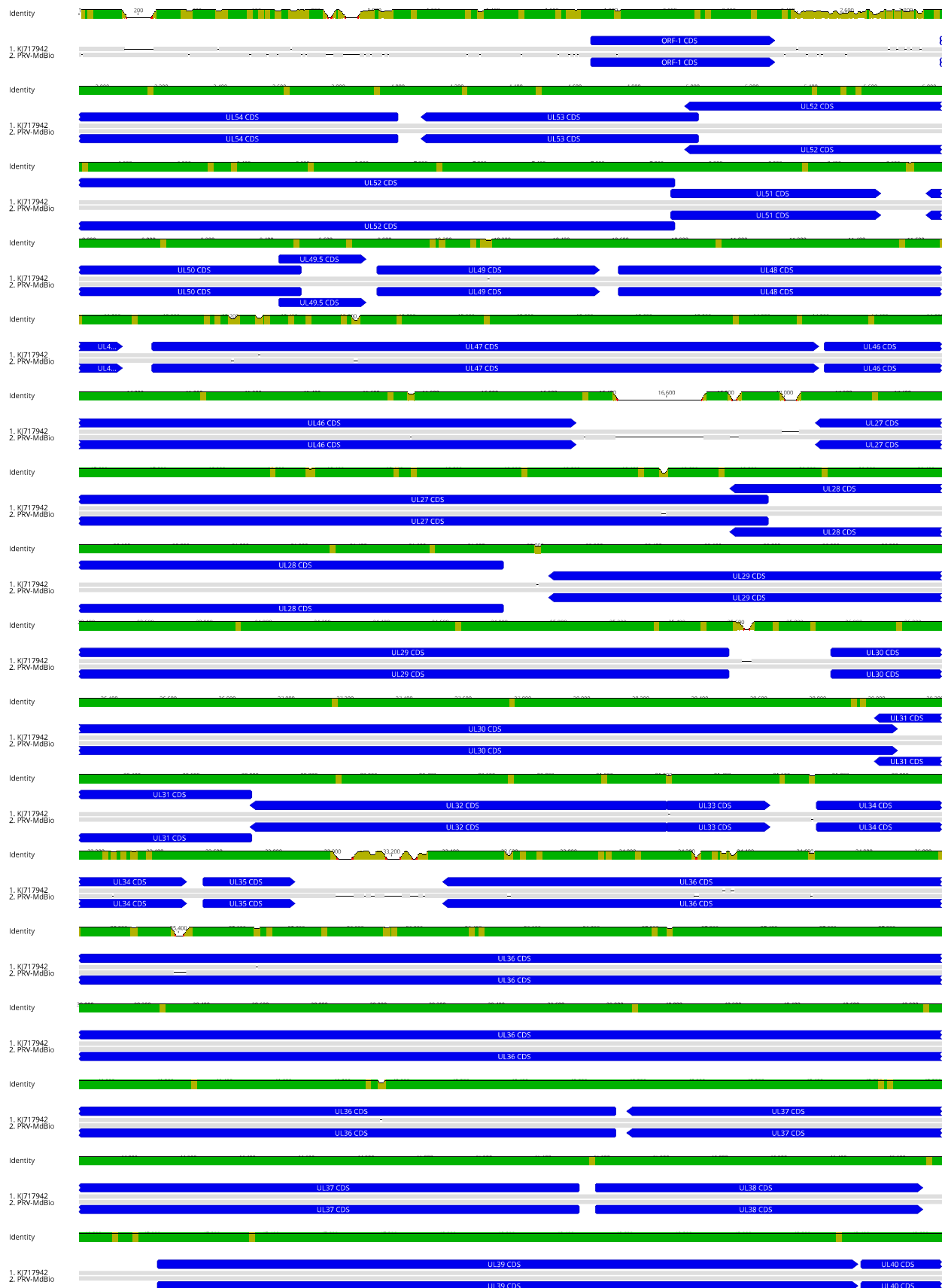

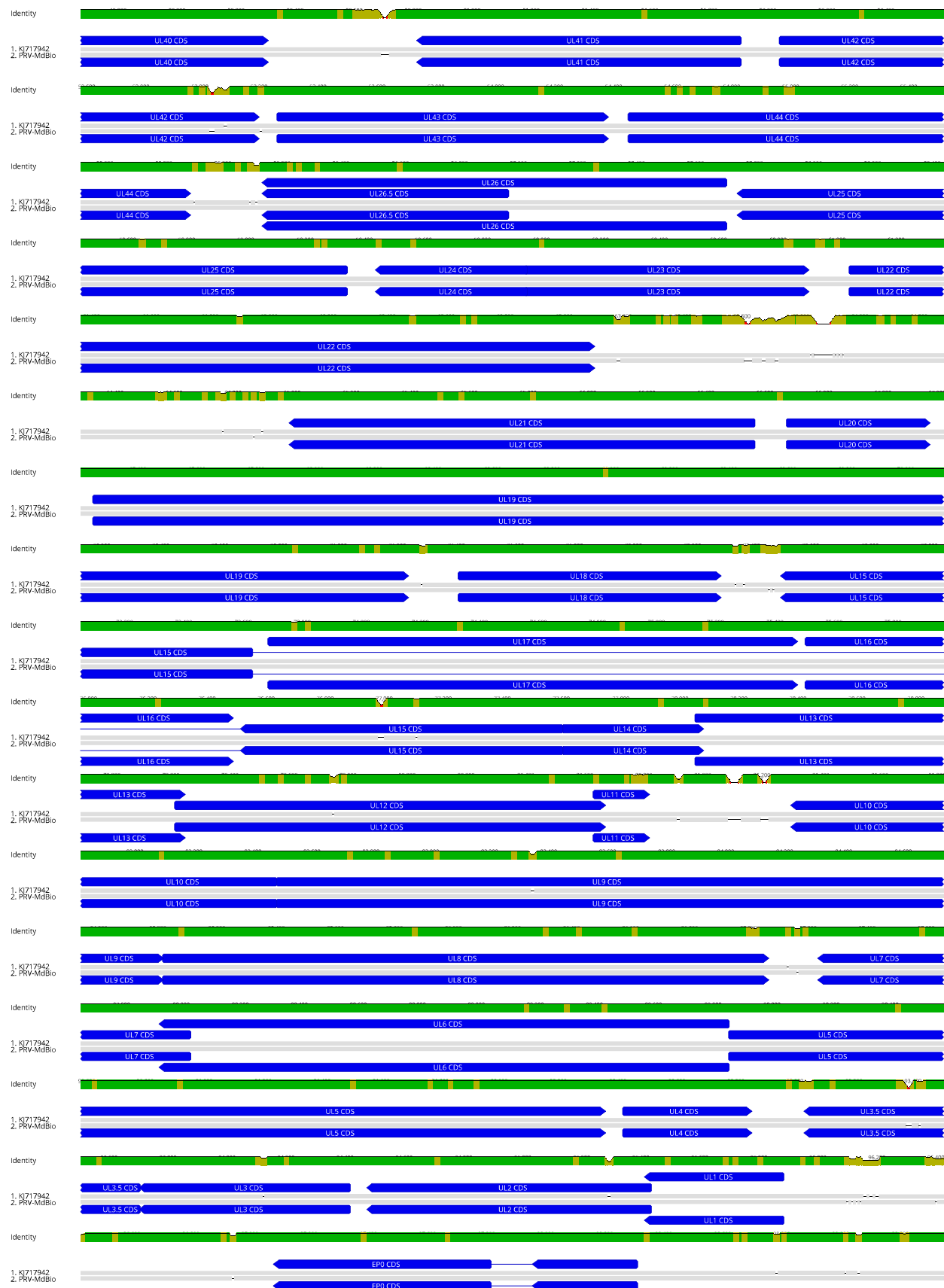

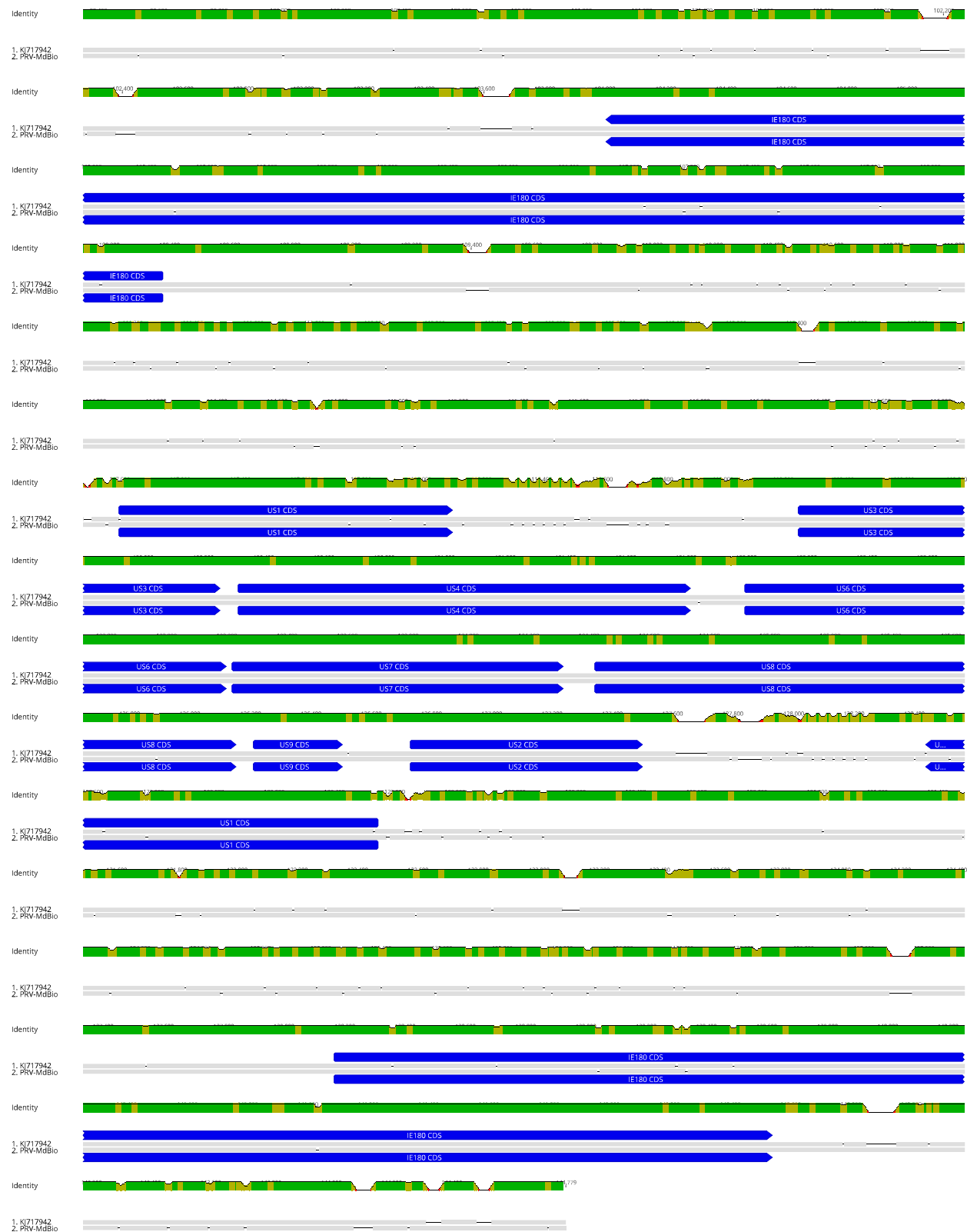

Supplement: Supplementary 4 — Additional file 4: horizontal chart representation of the number of SNVs and INDELs within the MdBio and Kaplan, and the MdBio and Ea strains. This horizontal bar chart indicates the total number of the SNVs and INDELs, which are different between the PRV-MdBio and two other viral strains (strains Ka and Ea). Panel A: blue color was used for labeling the number of SNVs within the ORFs and magenta for the INDELs. Panel B: blue color represents the normalized number of SNVs within the ORFs, while magenta was used for labeling the INDELs. [file 1806842.f4.pdf]
