# Supplementary material for: Analysis of the Complete Genome Sequence of a Novel, Pseudorabies Virus Strain Isolated in Southeast Europe
Source: Can J Infect Dis Med Microbiol. 2019 Apr 4;2019:1806842. doi: 10.1155/2019/1806842 (PMC6476139; doi:10.1155/2019/1806842)
Supplement: Supplementary 6 — Additional file 6: sequence comparison of the strain MdBio and Kaplan. This figure shows the sequence alignment of the strain MdBio and Kaplan. The genome sequences are represented by grey. The single-nucleotide variants are labeled by vertical, thin black lines within the genome. The thick black regions within the genome sequences are the insertions, whereas the deletions are represented by horizontal black lines. The protein-coding sequences of the genes are indicated by blue arrows. The histogram above the genomes shows the differences between the two sequences by yellow, while the identical parts are labeled by green. [file 1806842.f6.pdf]

A	MdBio-Kaplan		MdBio-Ea	
Gene	Total number of SNVs	Total number of INDELs	Total number of SNVs	Total number of INDELs
orf-1	9	1	30	1
ul54	6	0	29	0
ul53	4	0	23	0
ul52	16	0	53	6
ul51	2	0	18	4
ul50	4	0	16	0
ul49.5	4	0	13	0
ul49	11	1	15	4
ul48	6	0	19	0
ul47	25	3	66	4
ul46	7	1	73	4
ul27	19	1	41	2
ul28	6	0	22	2
ul29	8	0	33	3
ul30	10	0	27	0
ul31	0	0	12	0
ul32	6	0	14	1
ul33	1	1	7	1
ul34	11	1	25	0
ul35	0	0	2	0
ul36.5	6	5	92	11
ul36	62	8	370	30
ul37	4	0	48	0
ul38	0	0	15	0
ul39	4	0	28	1
ul40	0	0	4	0
ul41	2	0	19	0
ul42	26	2	15	2
ul43	2	0	8	0
ul44	22	0	53	1
ul26.5	8	0	13	3
ul26	10	0	16	3
ul25	10	0	14	2
ul24	4	0	5	0
ul23	6	0	5	0
ul22	12	0	4	1
ul21	6	0	31	2
ul20	0	0	13	2
ul19	8	0	27	0
ul18	0	0	8	0
ul17	10	0	35	2
ul16	2	0	36	0
ul15	4	2	45	5
ul14	2	0	8	0
ul13	4	0	29	1
ul12	17	1	31	2
ul11	10	0	4	0
ul10	2	0	19	0
ul9	11	1	26	1
ul8	18	0	45	1
ul7	2	0	11	0
ul6	6	0	6	4
ul5	14	0	3	2
ul4	0	0	4	0
ul3.5	22	2	15	2
ul3	5	1	10	1
ul2	5	1	6	2
ul1	8	0	17	0
ep0	4	0	15	2
ie180	37	7	54	12
us1	28	4	24	3
us3	10	0	22	0
us4	14	0	13	0
us6	0	0	15	1
us7	6	0	35	3
us8	21	1	29	2
us9	2	0	9	0
us2	2	0	18	0

B	MdBio-Kaplan		MdBio-Ea	
Gene	Total number of SNVs/ Length of ORF	Total number of INDELs/ Length of ORF	Total number of SNVs/ Length of ORF	Total number of INDELs/ Length of ORF
orf-1	0,0144	0,0016	0,0481	0,0016
ul54	0,0055	0,0000	0,0267	0,0000
ul53	0,0043	0,0000	0,0245	0,0000
ul52	0,0055	0,0000	0,0183	0,0021
ul51	0,0028	0,0000	0,0253	0,0056
ul50	0,0050	0,0000	0,0198	0,0000
ul49.5	0,0135	0,0000	0,0438	0,0000
ul49	0,0147	0,0013	0,0200	0,0053
ul48	0,0048	0,0000	0,0153	0,0000
ul47	0,0111	0,0013	0,0293	0,0018
ul46	0,0034	0,0005	0,0351	0,0019
ul27	0,0069	0,0004	0,0148	0,0007
ul28	0,0028	0,0000	0,0102	0,0009
ul29	0,0023	0,0000	0,0093	0,0008
ul30	0,0032	0,0000	0,0086	0,0000
ul31	0,0000	0,0000	0,0147	0,0000
ul32	0,0042	0,0000	0,0099	0,0007
ul33	0,0029	0,0029	0,0201	0,0029
ul34	0,0139	0,0013	0,0317	0,0000
ul35	0,0000	0,0000	0,0064	0,0000
ul36.5	0,0037	0,0030	0,0560	0,0067
ul36	0,0066	0,0009	0,0397	0,0032
ul37	0,0014	0,0000	0,0174	0,0000
ul38	0,0000	0,0000	0,0136	0,0000
ul39	0,0017	0,0000	0,0118	0,0004
ul40	0,0000	0,0000	0,0044	0,0000
ul41	0,0018	0,0000	0,0173	0,0000
ul42	0,0225	0,0017	0,0130	0,0017
ul43	0,0018	0,0000	0,0071	0,0000
ul44	0,0152	0,0000	0,0367	0,0007
ul26.5	0,0096	0,0000	0,0155	0,0036
ul26	0,0063	0,0000	0,0102	0,0019
ul25	0,0062	0,0000	0,0087	0,0012
ul24	0,0078	0,0000	0,0097	0,0000
ul23	0,0062	0,0000	0,0052	0,0000
ul22	0,0058	0,0000	0,0019	0,0005
ul21	0,0038	0,0000	0,0196	0,0013
ul20	0,0000	0,0000	0,0267	0,0041
ul19	0,0020	0,0000	0,0068	0,0000
ul18	0,0000	0,0000	0,0090	0,0000
ul17	0,0045	0,0000	0,0159	0,0009
ul16	0,0011	0,0000	0,0201	0,0000
ul15	0,0041	0,0020	0,0456	0,0051
ul14	0,0042	0,0000	0,0167	0,0000
ul13	0,0033	0,0000	0,0242	0,0008
ul12	0,0117	0,0007	0,0213	0,0014
ul11	0,0521	0,0000	0,0208	0,0000
ul10	0,0017	0,0000	0,0161	0,0000
ul9	0,0043	0,0004	0,0103	0,0004
ul8	0,0088	0,0000	0,0219	0,0005
ul7	0,0025	0,0000	0,0137	0,0000
ul6	0,0031	0,0000	0,0031	0,0021
ul5	0,0056	0,0000	0,0012	0,0008
ul4	0,0000	0,0000	0,0091	0,0000
ul3.5	0,0326	0,0030	0,0222	0,0030
ul3	0,0070	0,0014	0,0140	0,0014
ul2	0,0053	0,0011	0,0063	0,0021
ul1	0,0170	0,0000	0,0361	0,0000
ep0	0,0037	0,0000	0,0137	0,0018
ie180	0,0085	0,0016	0,0124	0,0028
us1	0,0256	0,0037	0,0219	0,0027
us3	0,0100	0,0000	0,0219	0,0000
us4	0,0093	0,0000	0,0087	0,0000
us6	0,0000	0,0000	0,0125	0,0008
us7	0,0055	0,0000	0,0319	0,0027
us8	0,0121	0,0006	0,0167	0,0012
us9	0,0067	0,0000	0,0303	0,0000
us2	0,0026	0,0000	0,0233	0,0000

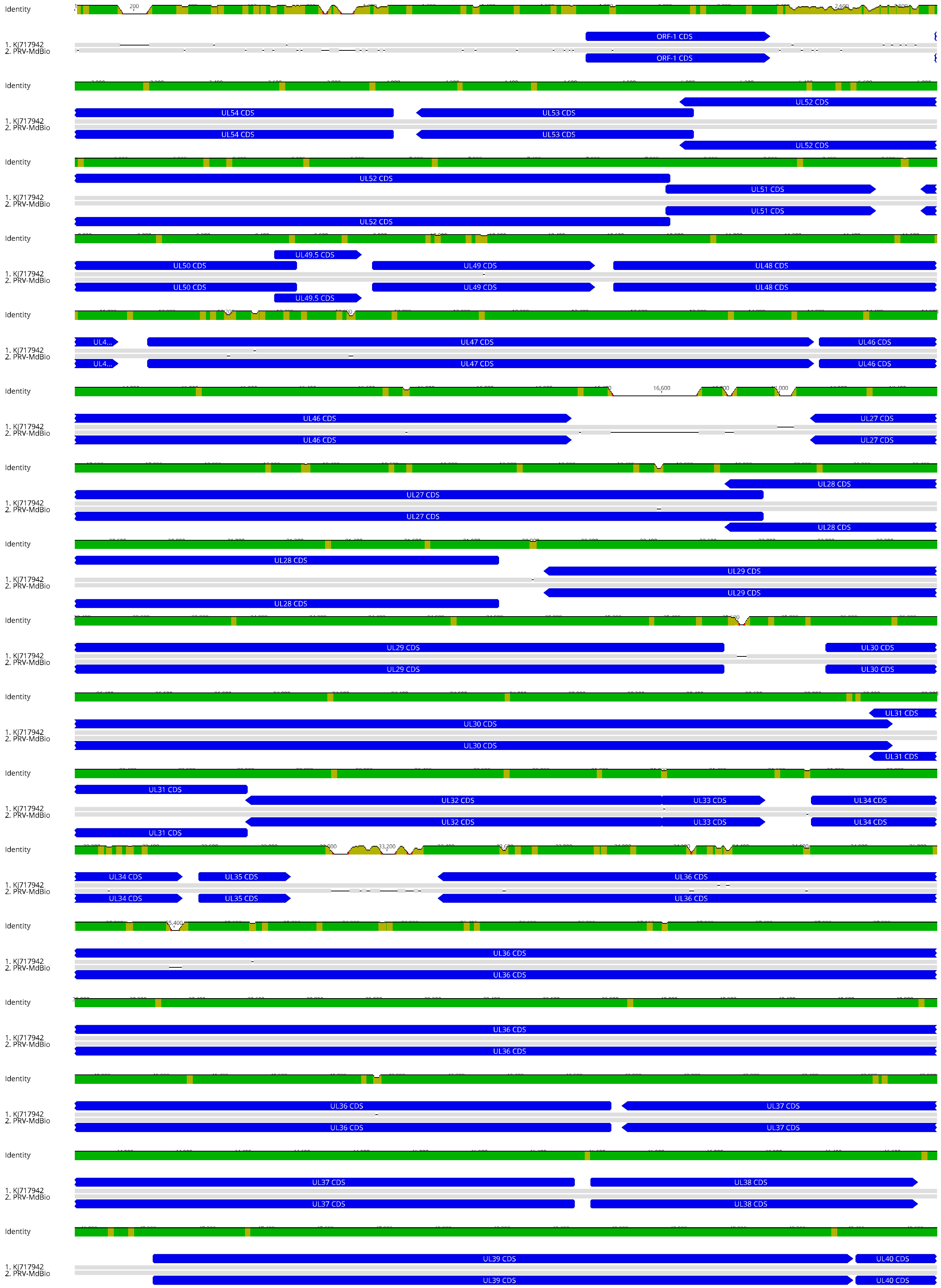
Identity
1. KJ717942
2. PRV-MdBio
ORF-1 CDS
UL54 CDS
UL53 CDS
UL52 CDS
UL51 CDS
UL50 CDS
UL49.5 CDS
UL49 CDS
UL48 CDS
UL4...
UL47 CDS
UL46 CDS
UL27 CDS
UL28 CDS
UL29 CDS
UL30 CDS
UL31 CDS
UL32 CDS
UL33 CDS
UL34 CDS
UL35 CDS
UL36 CDS
UL37 CDS
UL38 CDS
UL39 CDS
UL40 CDS
200
16,600
33,200

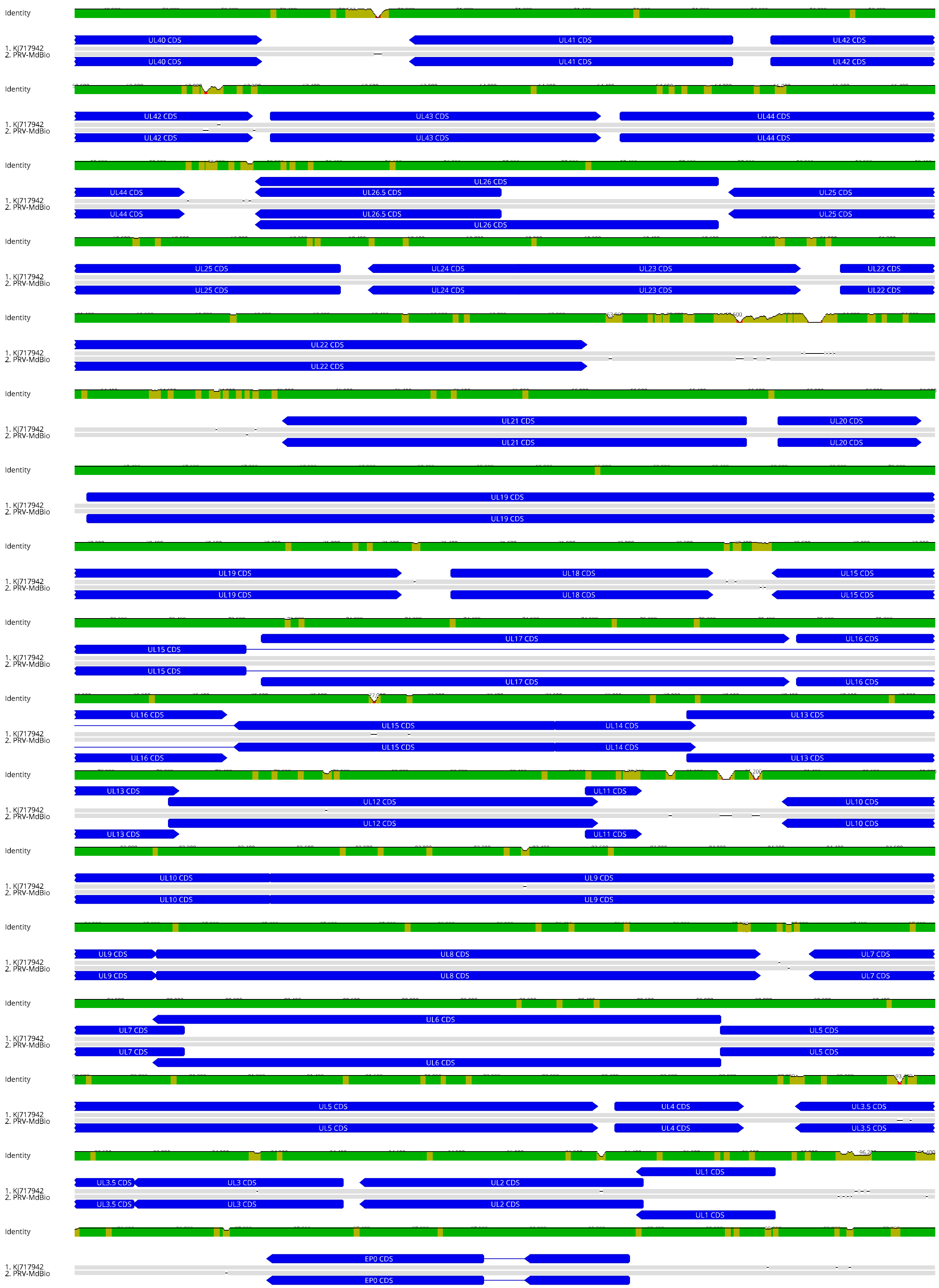

Identity
1. KJ717942
2. PRV-MdBio
UL40 CDS
UL41 CDS
UL42 CDS
UL43 CDS
UL44 CDS
UL26 CDS
UL26.5 CDS
UL25 CDS
UL24 CDS
UL23 CDS
UL22 CDS
UL21 CDS
UL20 CDS
UL19 CDS
UL18 CDS
UL15 CDS
UL17 CDS
UL16 CDS
UL14 CDS
UL13 CDS
UL12 CDS
UL11 CDS
UL10 CDS
UL9 CDS
UL8 CDS
UL7 CDS
UL6 CDS
UL5 CDS
UL4 CDS
UL3.5 CDS
UL3 CDS
UL2 CDS
UL1 CDS
EP0 CDS

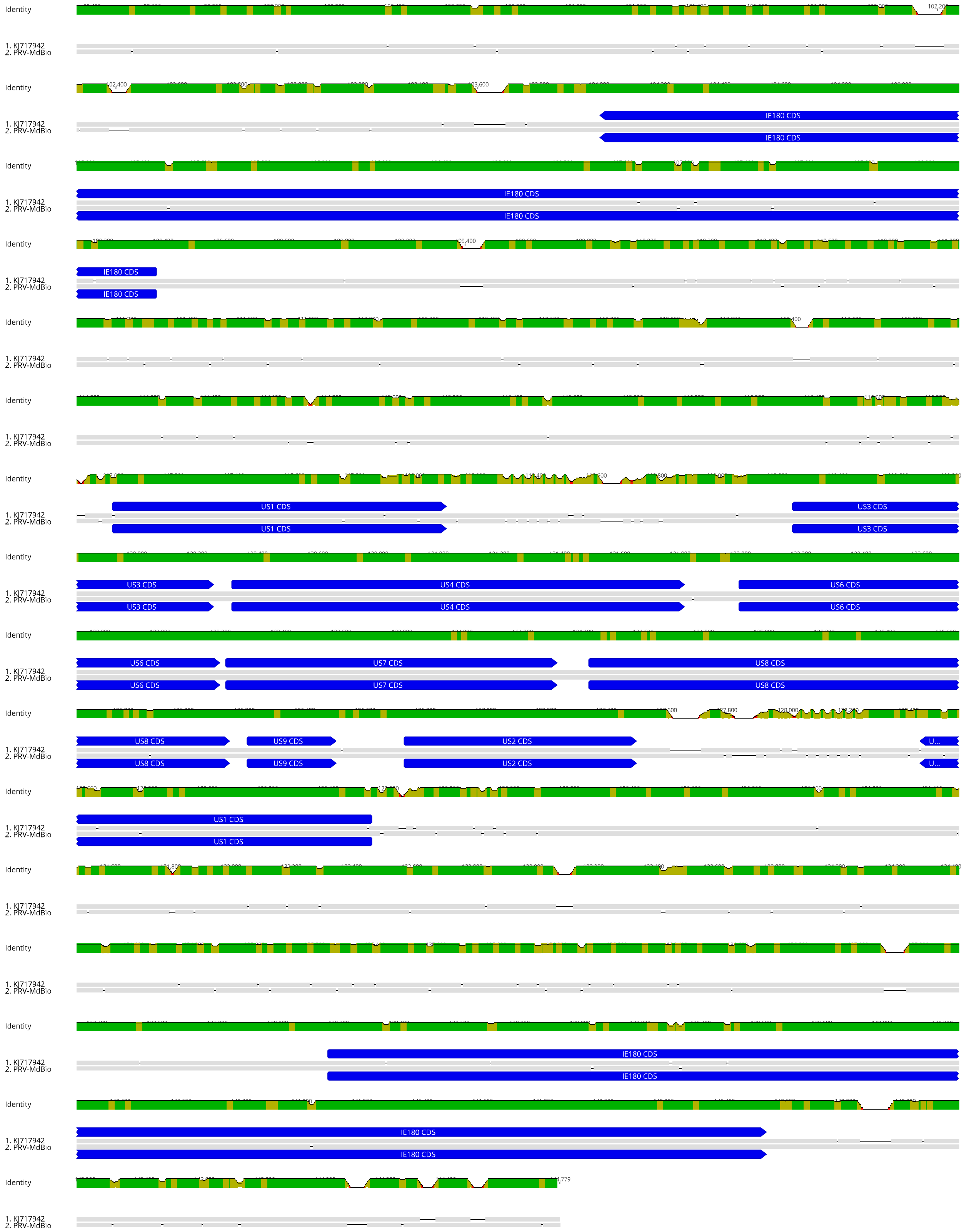
Identity
1. KJ717942
2. PRV-MdBio
IE180 CDS
US1 CDS
US3 CDS
US4 CDS
US6 CDS
US7 CDS
US8 CDS
US9 CDS
US2 CDS
U...

Time	Ka 0.1 pfu/cell		MdBio 0.1 pfu/cell		Ka 10 pfu/cell		MdBio 10 pfu/cell	
	Mean	SE	Mean	SE	Mean	SE	Mean	SE
**4h**	1.03E+01	8.39E-01	6.67E+00	1.64E+00	6.07E+02	3.89E+01	5.73E+02	4.86E+01
**8h**	1.70E+03	3.48E+02	1.35E+03	1.96E+02	6.50E+05	1.30E+05	1.41E+06	5.20E+05
**12h**	6.07E+03	5.40E+02	5.97E+03	7.46E+02	3.07E+07	4.76E+06	2.93E+07	3.02E+06
**18h**	3.73E+05	1.19E+05	8.60E+04	4.84E+03	4.90E+08	5.78E+07	4.50E+08	7.37E+07
**24h**	7.77E+05	1.04E+05	7.67E+05	7.13E+04	1.26E+08	2.13E+07	8.93E+07	3.02E+06

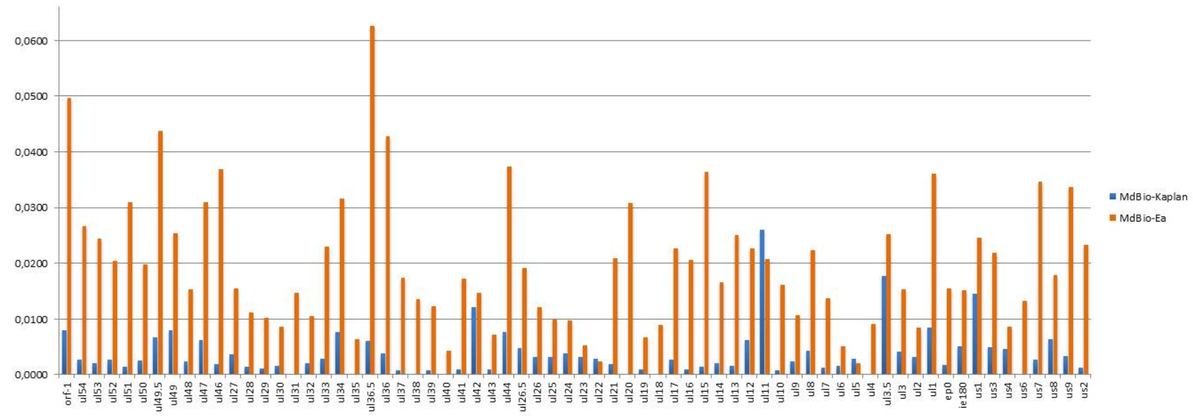
0,0600
0,0500
0,0400
0,0300
0,0200
0,0100
0,0000
orf-1
ul54
ul53
ul52
ul51
ul50
ul49.5
ul49
ul48
ul47
ul46
ul27
ul28
ul29
ul30
ul31
ul32
ul33
ul34
ul35
ul36.5
ul36
ul37
ul38
ul39
ul40
ul41
ul42
ul43
ul44
ul26.5
ul26
ul25
ul24
ul23
ul22
ul21
ul20
ul19
ul18
ul17
ul16
ul15
ul14
ul13
ul12
ul11
ul10
ul9
ul8
ul7
ul6
ul5
ul4
ul3.5
ul3
ul2
ul1
ep0
ie180
us1
us3
us4
us6
us7
us8
us9
us2
MdBio-Kaplan
MdBio-Ea

Name	Sequence (5'-3')
**ul23 fw**	ATGACGGTCGTCTTTGACCGCCAC
**ul23 rev**	CGCTGATGTCCCCGACGATGAA
**ul29 fw**	CTGATCCTGCGCTACTGCG
**ul29 rev**	ACTGCATCGTGATCCCCG
**ul44 fw**	TCGTGAGCAGCATGATCGT
**ul44 rev**	GTCGCCATGATGACCAGC
**ie180 fw**	CATCGTGCTGGACACCATCGAG
**ie180 rev**	ACGTAGACGTGGTAGTCCCCCA

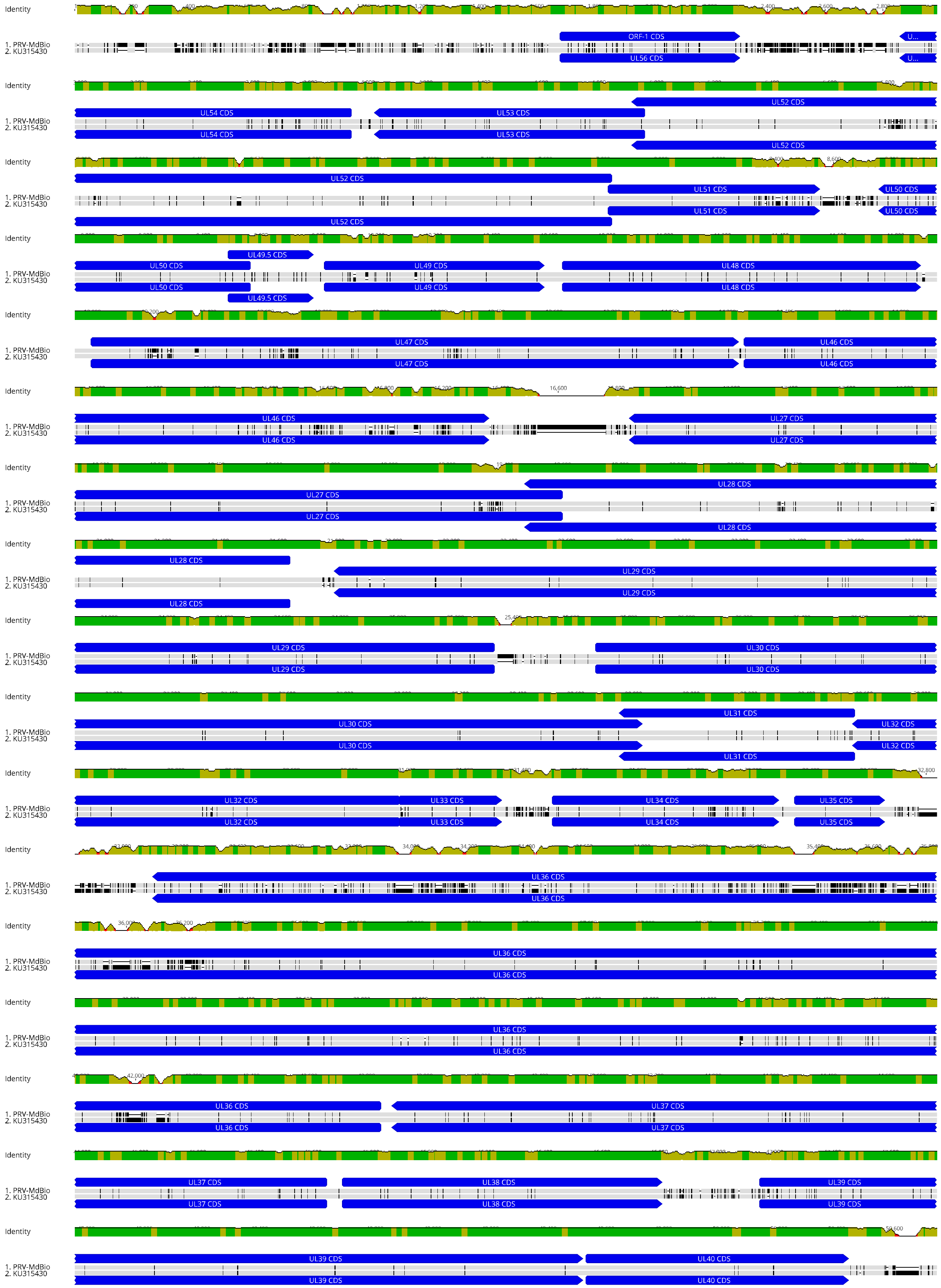
Identity
1. PRV-MdBio
2. KU315430
ORF-1 CDS
UL56 CDS
UL54 CDS
UL53 CDS
UL52 CDS
UL51 CDS
UL50 CDS
UL49.5 CDS
UL49 CDS
UL48 CDS
UL47 CDS
UL46 CDS
UL27 CDS
UL28 CDS
UL29 CDS
UL30 CDS
UL31 CDS
UL32 CDS
UL33 CDS
UL34 CDS
UL35 CDS
UL36 CDS
UL37 CDS
UL38 CDS
UL39 CDS
UL40 CDS

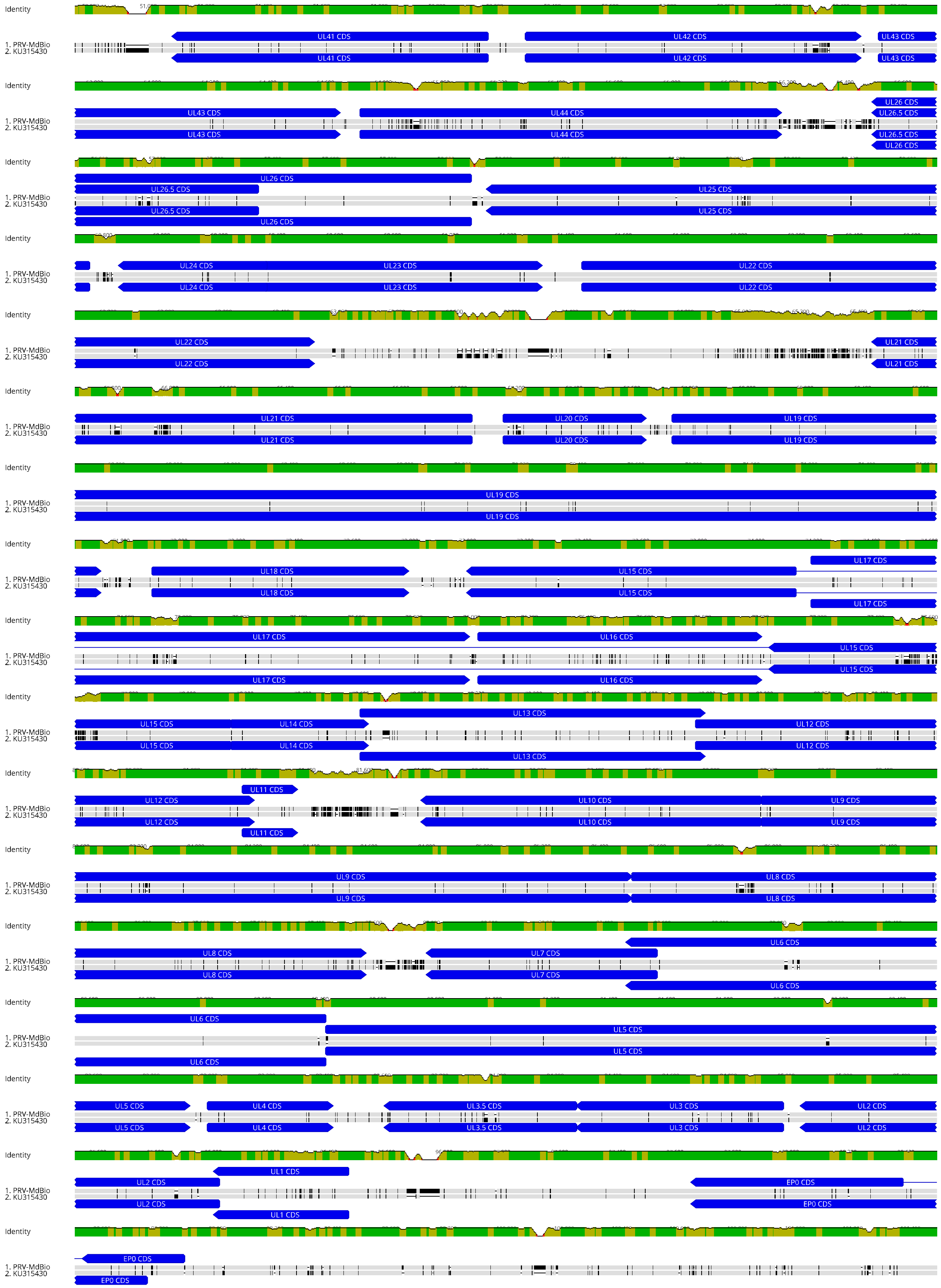
Identity
1. PRV-MdBio
2. KU315430
UL41 CDS
UL42 CDS
UL43 CDS
UL44 CDS
UL26 CDS
UL26.5 CDS
UL25 CDS
UL24 CDS
UL23 CDS
UL22 CDS
UL21 CDS
UL20 CDS
UL19 CDS
UL18 CDS
UL15 CDS
UL17 CDS
UL16 CDS
UL14 CDS
UL13 CDS
UL12 CDS
UL11 CDS
UL10 CDS
UL9 CDS
UL8 CDS
UL7 CDS
UL6 CDS
UL5 CDS
UL4 CDS
UL3.5 CDS
UL3 CDS
UL2 CDS
UL1 CDS
EP0 CDS

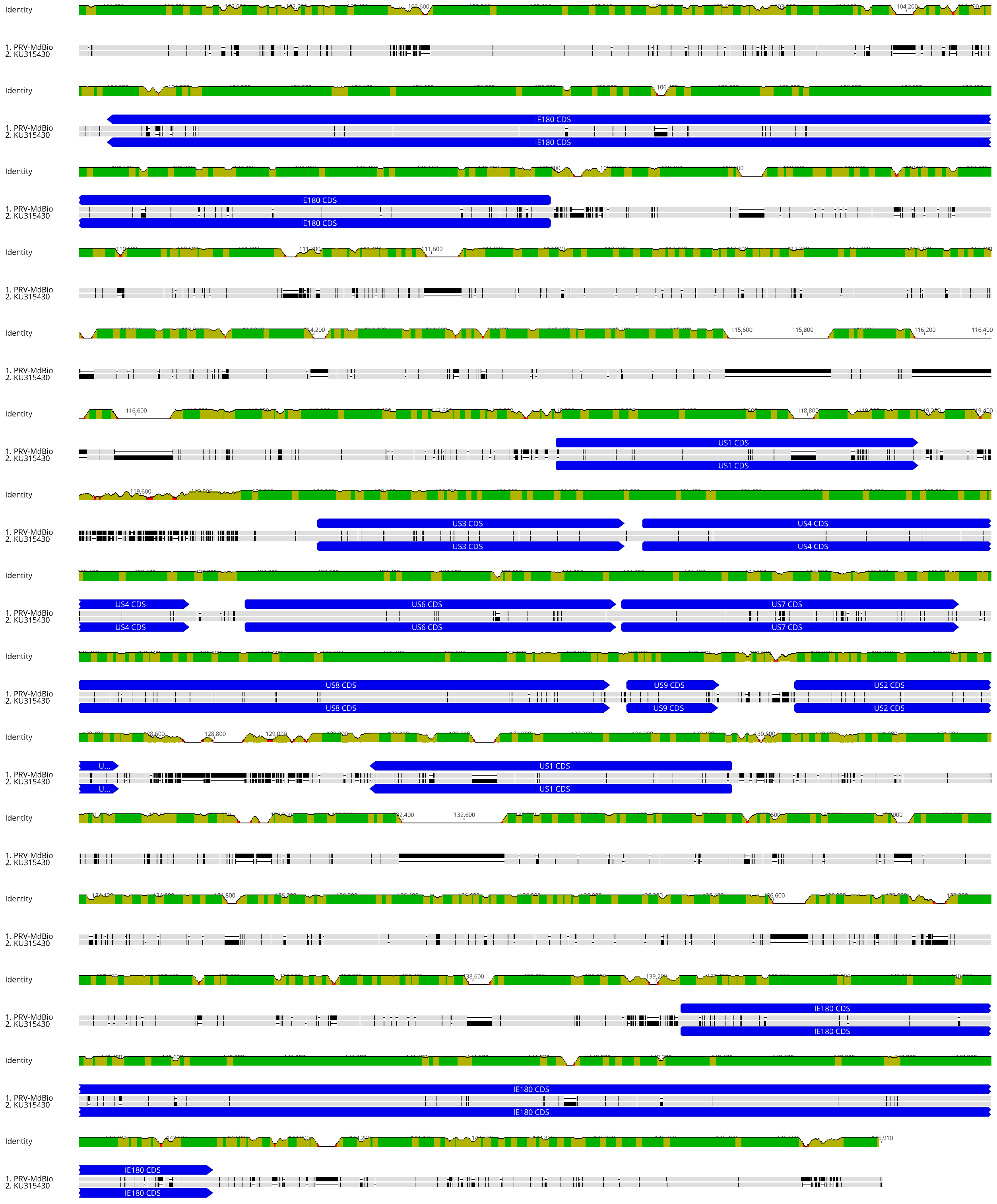
Identity
1. PRV-MdBio
2. KU315430
IE180 CDS
US1 CDS
US3 CDS
US4 CDS
US6 CDS
US7 CDS
US8 CDS
US9 CDS
US2 CDS
115,600
115,800
116,200
116,400
116,600
128,800
129,000
132,600
